# Supplementary material for: ICAM-1 as a biomarker of efficacy for oncolytic coxsackievirus A21 in patient-derived Ewing sarcoma models
Source: Mol Ther Oncol. 2026 Oct 14;34(4):201367. doi: 10.1016/j.omton.2026.201367 (PMC13755567; doi:10.1016/j.omton.2026.201367)
Supplement: Supplementary file 1 [file mmc1.pdf]

OMTON, Volume *34*

Supplemental information

ICAM-1 as a biomarker of efficacy for oncolytic coxsackievirus A21 in patient-derived Ewing sarcoma models

Richard T. Baugh, Tyler Barr, Elizabeth A. Roundhill, Fay Ismail, Milene Volpato, Graham P. Cook, Susan A. Burchill, and Fiona Errington-Mais

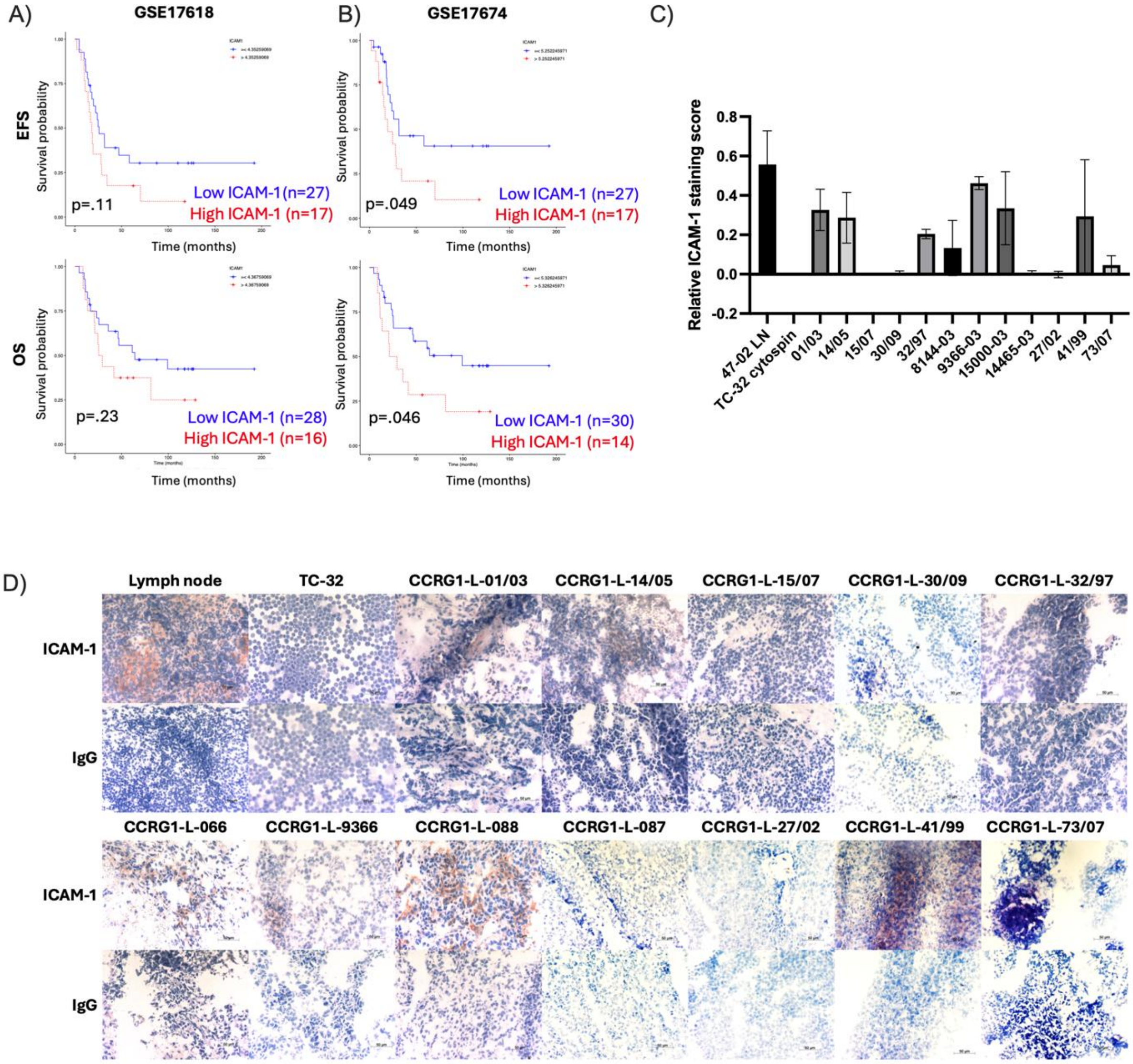

Figure S1. ICAM-1 expression in EWS. **A)** Kaplan-Meier event-free survival (EFS) and overall survival (OS) curves of high and low ICAM-1 expression from publicly available data sets GSE17618 and **B)** GSE17674. **C)** Quantification of IHC for ICAM-1. ICAM-1 staining was quantified using ImageJ analysis, with ICAM-1 score of DAB signal normalised to H&E nuclear staining for each image. N≥3 representative images taken for each section on 40x magnification. ICAM-1 scores were normalised by subtracting mean staining score from isotype-stained samples. **D)** Representative examples of ICAM-1 staining in EWS tissue by IHC. Isotype control (IgG2b) was used as a negative control for each sample. Human Lymph node tissue was used as a positive control and a cytospin of TC-32 cells used as a negative control. Representative images are shown at 40x magnification.

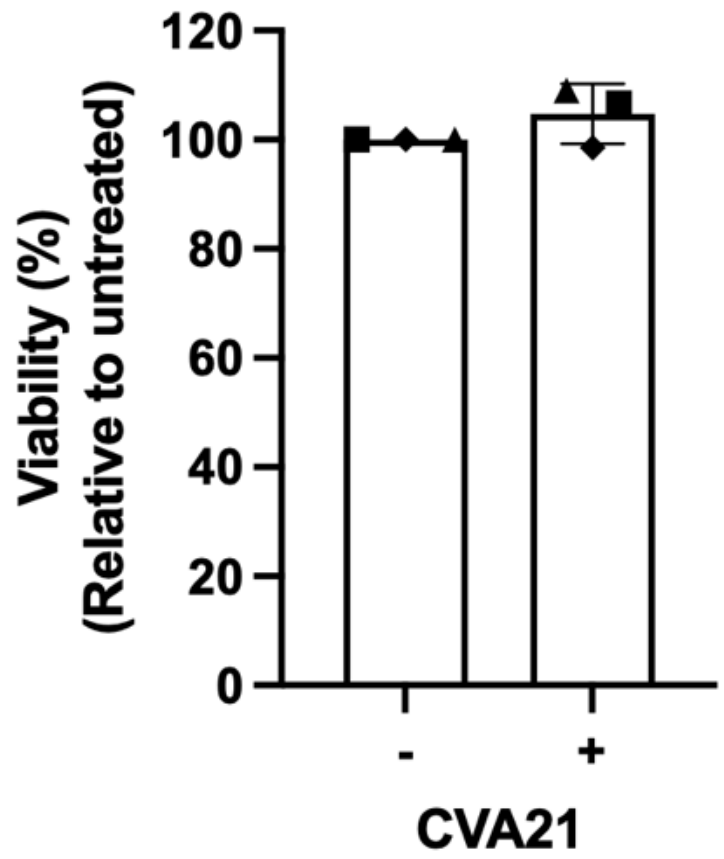

Figure S2. Short-term CVA21 infection of PDES has no oncolytic effect. A) PDES (n=3) were infected with CVA21 (MOI 2.5 – equivalent dose used in CVA21-stimulated PBMC assays; 5 hours). Cell viability was measured by CellTitre Glo and data shows the mean percentage viability of cells, relative to the untreated control (± SD). A paired t-test was performed.

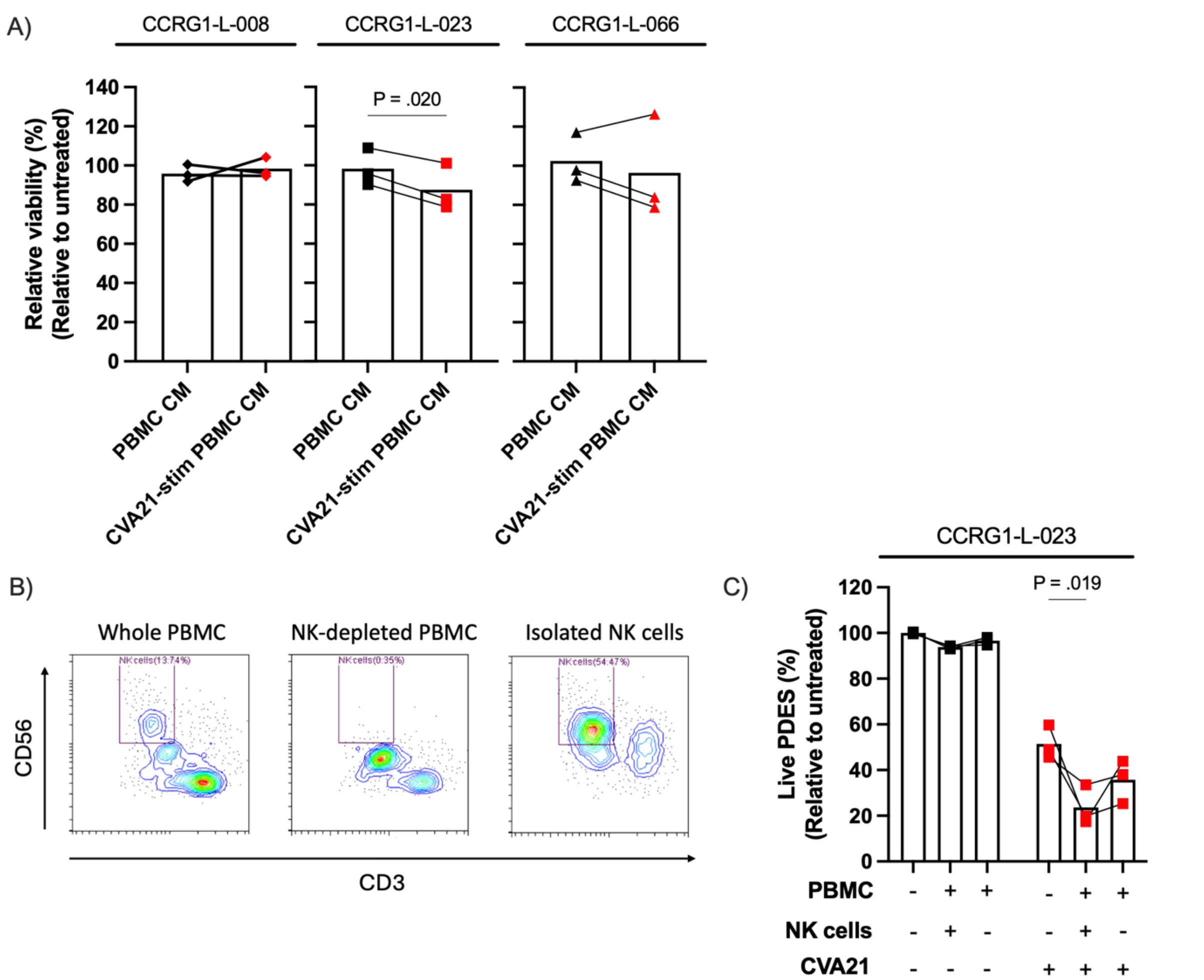

Figure S3. Mechanisms of immune-mediated killing of PDES. A) Percentage cell viability of PDES (n=3) treated with PBMC CM or CVA21-stimulated PBMC conditioned media (1:1 RPMI:CM). For PBMC CM, n=3 donors; CVA21 stimulation with MOI 0.1 for 48 hours. Cell viability was measured by CellTitre Glo 48 hours post treatment. Data is presented as mean percentage viability of cells relative to the untreated control (± SD). Paired t-tests were performed. **B)** Representative flow cytometry plots following NK cell depletion from whole PBMC. PBMC were stained with anti-CD45, anti-CD56 and anti-CD3. Plots represent live CD45$^+$ population. **C)** Percentage live PDES cells infected with CVA21 alone, or in co-culture with whole PBMC or NK-depleted PBMC. Adherent PDES were infected (or left untreated) with CVA21 (MOI 0.5) for 2 hours before co-culture with unstimulated PBMC ± NK cells for 48 hours (n=1; n=3 PBMC donors) at a 10:1 PBMC: PDES ratio. Cell viability was measured using LIVE/DEAD flow cytometry and data is presented as mean percentage live PDES (CD45$^-$) relative to

PDES in the absence of PBMC; each line represents a different PBMC donor. A two-way ANOVA with Šídák's post-hoc test was performed.

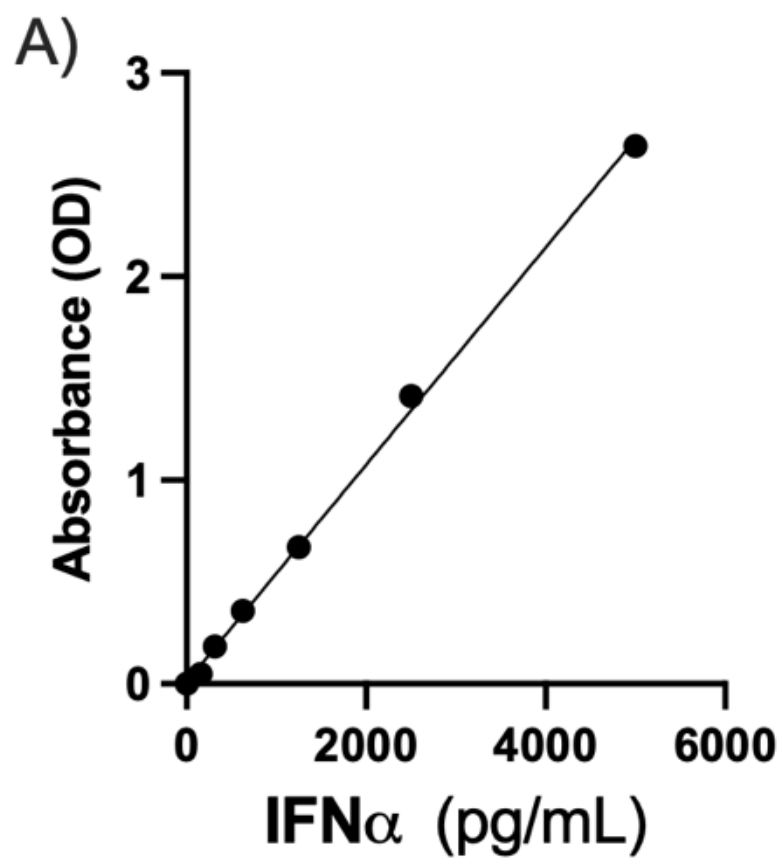

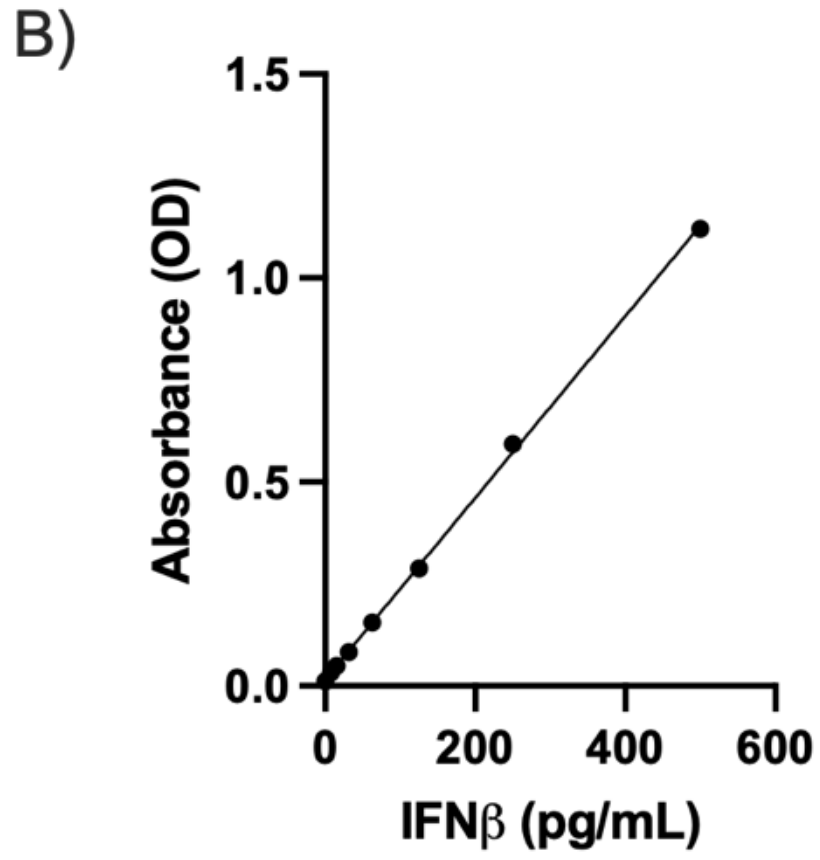

IFNα mean normalised OD values

	PDES		
MOI	CCRG-L-017	CCRG-L-023	CCRG-L-066
0	-98.67	-58.00	-111.33
0.1	-83.33	-98.00	-106.00
1	-107.33	-78.00	-122.67

IFNβ mean normalised OD values

	PDES		
MOI	CCRG-L-017	CCRG-L-023	CCRG-L-066
0	0.07	0.03	0.03
0.1	0.07	0.06	0.03
1	0.07	0.06	0.04

Figure S4. PDES do not produce type I IFN in response to CVA21. **A)** Standard curves and normalised optical density (OD) values from IFNα and **B)** IFNβ ELISAs. PDES (n=3) were infected with CVA21 at MOI 0, 0.1 and 1 for 48h before supernatant was collected for ELISA. OD readings normalised by subtracting blank well OD values. Data represents mean normalised OD from n=2 technical repeats.

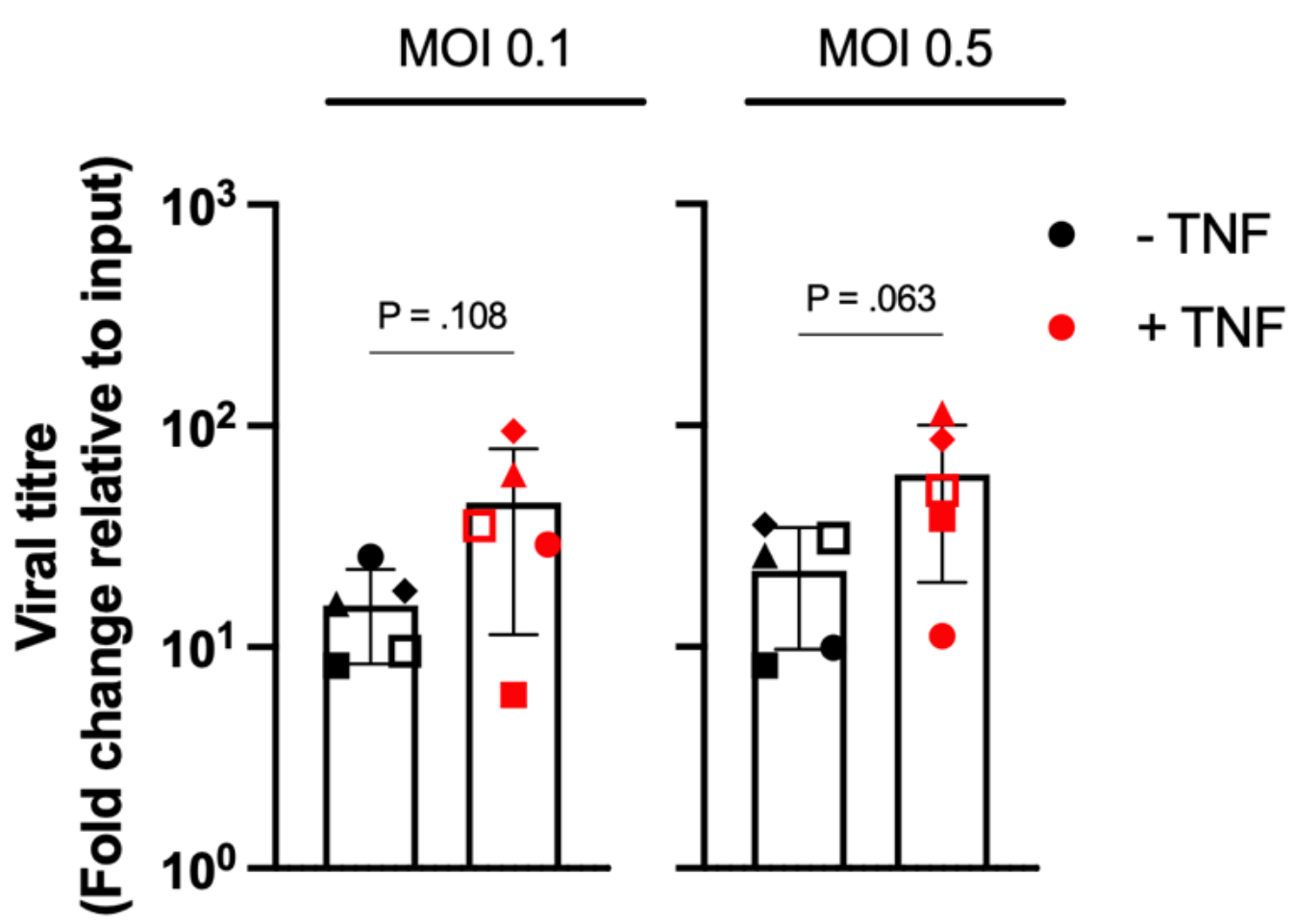

Figure S5. Fold change in CVA21 viral titre after PDES (n=5) were pre-treated with ± TNFα (5 ng/mL; 24 hours) before infection with CVA21 (MOI 0.1 and 0.5). Total cell supernatant and lysates were collected 16h post infection and CVA21 titres were measured by plaque assay. Mean fold change in viral titre compared to input virus titre for each PDES is shown. A paired t-test was performed.

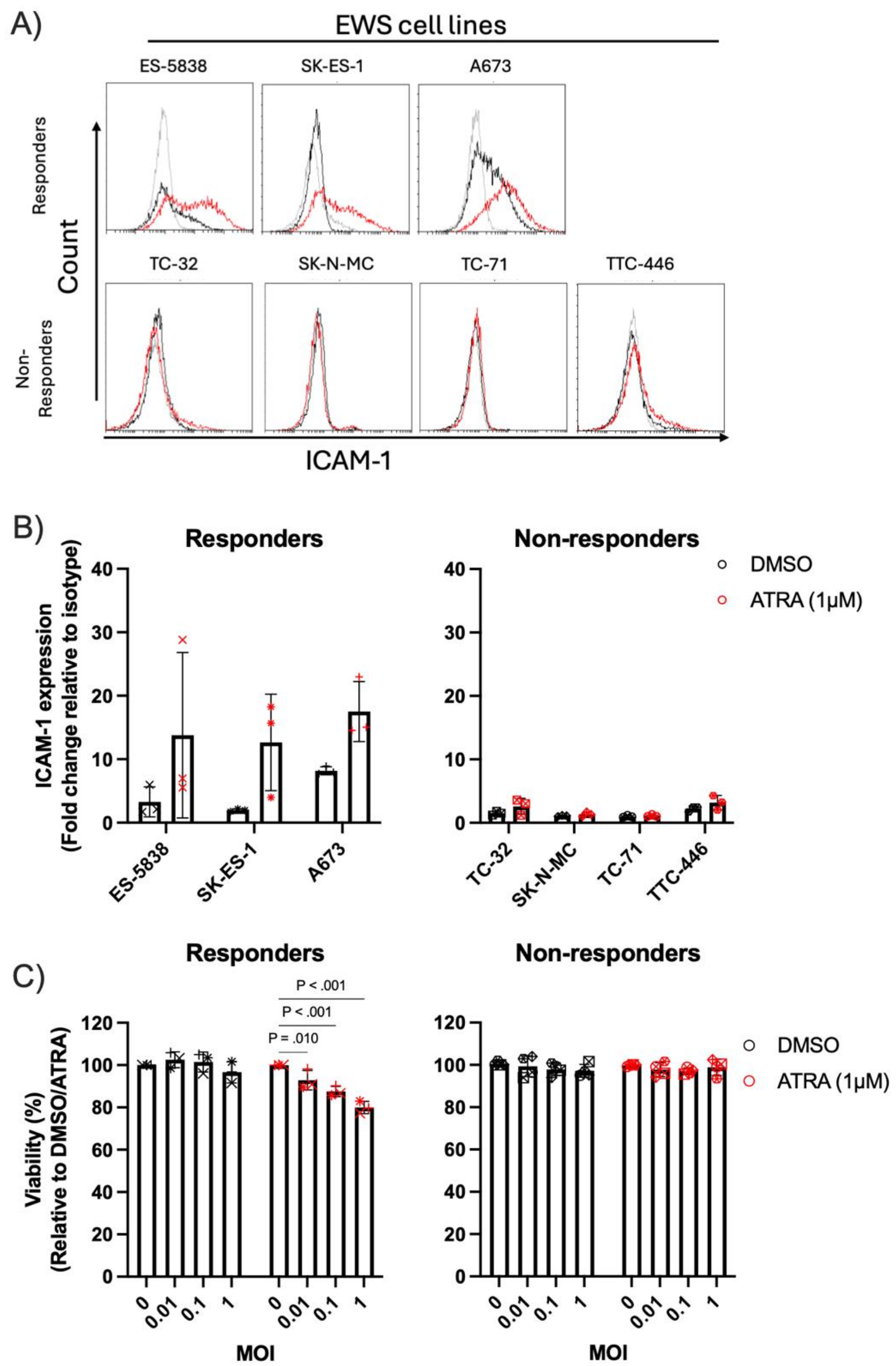

Figure S6. EWS cell lines display heterogeneous responses to ATRA-induced ICAM-1 expression and CVA21 susceptibility A) Representative flow cytometry histograms of EWS cell line surface ICAM-1 expression on control (DMSO; 72 hours; black line) and ATRA treated (1 μM; 72 hours; red line) cells compared to isotype control (grey line). **B)** Relative surface ICAM-1 expression on EWS cell lines (n=7) after treatment described in A). EWS cell lines were grouped as responders (ES-5838, SK-ES-1 and A673) and non-responders (TC-32, SK-N-MC, TC-71 and TTC-446). A two-way ANOVA with

Šídák's multiple comparisons test was performed. **C)** Percentage cell viability of EWS cell lines (n=7) following pre-treatment described in A) before infection with increasing doses of CVA21. Cell viability was measured by CellTitre Glo 48h post-infection. Data shows the mean percentage viability of cells relative to the DMSO or ATRA alone control (± SD). EWS cell lines were grouped as responders and non-responders in terms of ICAM-1 upregulation in A). A Two-way ANOVA with Dunnett's multiple comparisons test was performed.

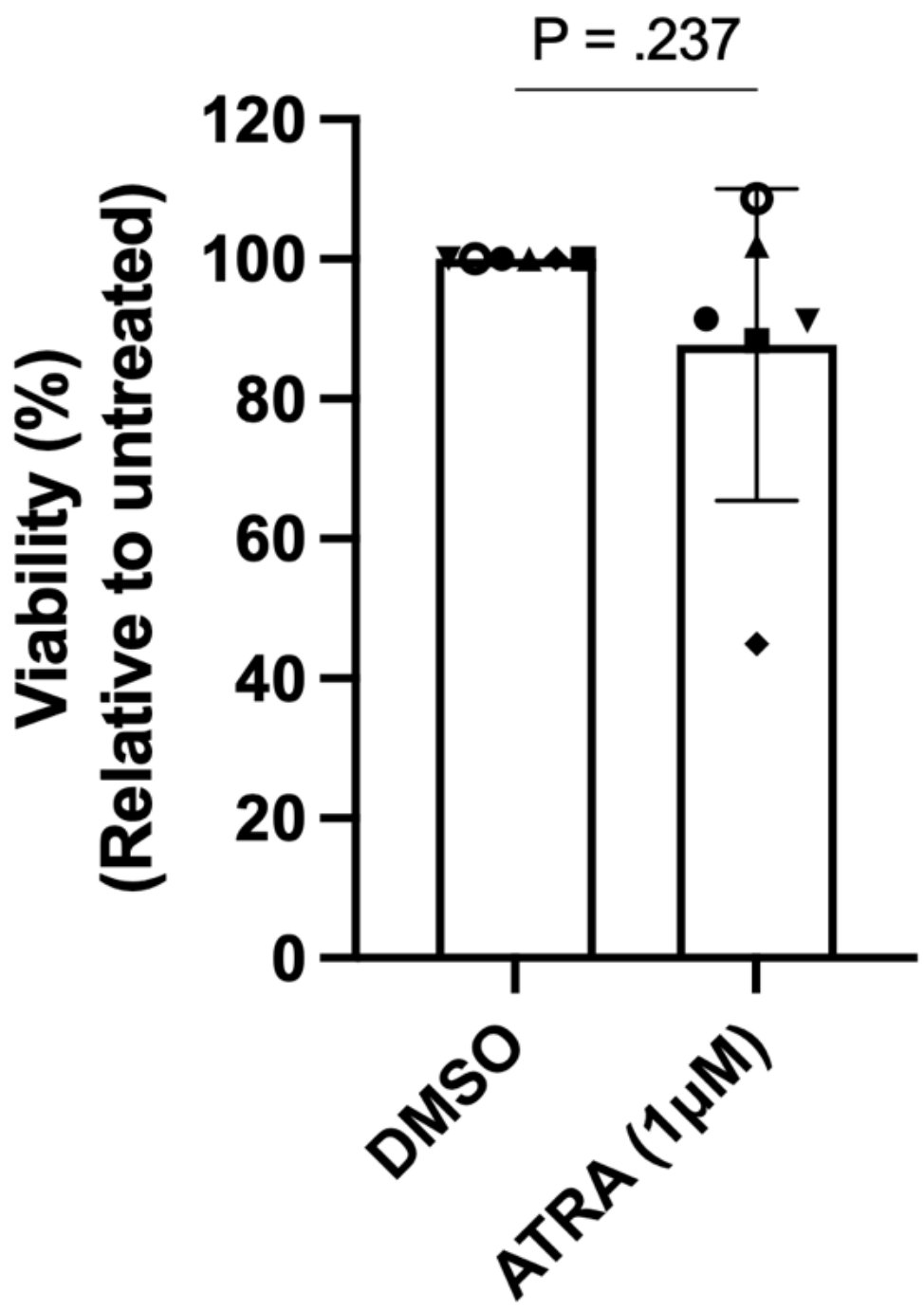

Figure S7. Cytostatic effects of ATRA on PDES. Relative viability of PDES (n=6) following treatment with 1 µM ATRA or DMSO for 72h. Cell viability was measured by CellTitre Glo 72 hours post treatment. Data is presented as mean percentage viability of PDES relative to the DMSO control. A paired t-test was performed.

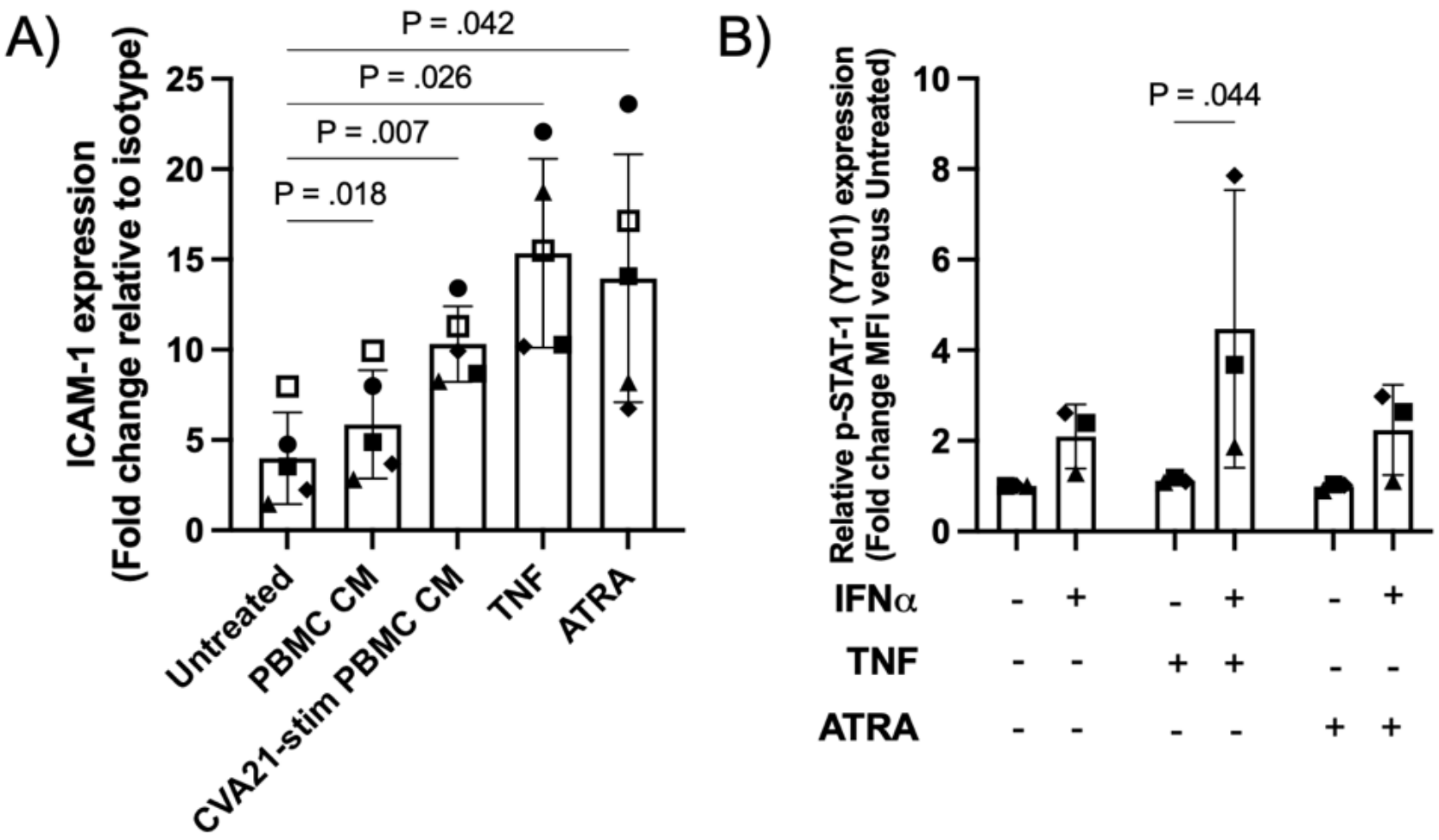

Figure S8. **Relative PDES surface ICAM-1 and intracellular phospho-STAT1 expression with different pre-treatment strategies**. **A)** Fold change in PDES (n=5) surface ICAM-1 expression following treatment with PBMC conditioned media (PBMC CM; 1:1 RPMI:CM; 24h), CVA21-stimulated PBMC conditioned media (CVA21-stim PBMC CM; 1:1 RPMI:CM; 24h), TNF (5 ng/mL; 24h) or ATRA (1μM; 72h). Data represents mean fold change in ICAM-1 expression relative to untreated control cells. PBMC CM was collected from n=3 individual donors. A one-way ANOVA with Dunnett's multiple comparisons test was performed. **B)** Fold change intracellular pSTAT-1 expression following pre-treatment with TNF (5 ng/mL; 24h) or ATRA (1μM; 72h) before treatment with IFNα (5 ng/mL; 30 min). Data represents mean fold change in pSTAT-1 expression relative to isotype control from n=3 PDES. A two-way ANOVA with Šídák's multiple comparisons test was performed.

Table S1. Summary of samples stained for ICAM-1 immunohistochemistry

Sample	Quality	ICAM-1 detected	Relative ICAM-1 score (±S.D)	Notes
Lymph node	+	+	0.56±0.17	Positive control
TC-32	+	-	0.00±0.00	Negative control
CCRG1-L-01/03	+	+	0.33±0.11	
CCRG1-L-14/05	+	+	0.29±0.13	
CCRG1-L-30/09	+	-		
CCRG1-L-32/97	+	-	0.20±0.02	
CCRG1-L-066	+	+	0.13±0.14	Matched PDES
CCRG1-L-9366	+	+	0.46±0.03	
CCRG1-L-088	+	+	0.33±0.19	Relapsed tumour<br>Matched PDES
CCRG1-L-087	+	-	0.01±0.01	Relapsed tumour<br>Matched PDES
CCRG1-L-27/02	+	-	0.00±0.02	
CCRG1-L-41/99	+	+	0.29±0.29	
CCRG1-L-73/07	+	-	0.05±0.05	
CCRG1-L-023	-	N/A		Poor tissue quality
CCRG1-L-075	-	N/A		Poor tissue quality
CCRG1-L-35/04	-	N/A		High non-specific staining
CCRG1-L-72/03	-	N/A		High non-specific staining
**Total**	**11/15**	**6/11**		
