# Supplementary material for: ICAM-1 as a biomarker of efficacy for oncolytic coxsackievirus A21 in patient-derived Ewing sarcoma models
Source: Mol Ther Oncol. 2026 Oct 14;34(4):201367. doi: 10.1016/j.omton.2026.201367 (PMC13755567; doi:10.1016/j.omton.2026.201367)
Supplement: Supplementary file 2 [file mmc2.pdf]

Molecular Therapy
Oncology
Original Article

ICAM-1 as a biomarker of efficacy for oncolytic coxsackievirus A21 in patient-derived Ewing sarcoma models

Richard T. Baugh,[1] Tyler Barr,[1] Elizabeth A. Roundhill,[1] Fay Ismail,[1] Milene Volpato,[1] Graham P. Cook,[1] Susan A. Burchill,[1] and Fiona Errington-Mais[1]

[1]Leeds Institute of Medical Research, University of Leeds, St James's University Hospital, Wellcome Trust Brenner Building, Beckett Street, Leeds LS9 7TF, UK

Ewing sarcoma (EWS) is a rare pediatric malignancy of the bone and soft tissue and requires better-tolerated and improved therapies. Oncolytic viruses (OVs) preferentially replicate in cancer cells, causing oncolysis, and activate both innate and adaptive anti-tumor immunity. Thus, OVs have the potential to generate long-lasting, systemic anti-cancer activity. Using a unique panel of patient-derived EWS cultures (PDES), recently derived from patient tumors at diagnosis and relapse, we have identified oncolytic coxsackievirus A21 (CVA21) as a candidate therapeutic OV in EWS, due to its high oncolytic activity and immune stimulation. PDES, unlike established EWS cell lines, expressed intercellular adhesion molecule-1 (ICAM-1), the CVA21 viral entry receptor, and were sensitive to CVA21-mediated oncolysis. Treatment with known ICAM-1 inducers, including tumor necrosis factor (TNF) and the clinically deliverable agent all-*trans* retinoic acid (ATRA), increased ICAM-1 expression and enhanced CVA21-induced oncolysis. Importantly, while CVA21 replication and oncolysis were inhibited by antiviral type I interferon, pre-treatment with ICAM-1 inducers (or CVA21-induced immune-mediated cytokines) overcame this inhibition. In addition to direct oncolysis, co-culture of CVA21-infected PDES with peripheral blood mononuclear cells (PBMCs) stimulated significant immune-mediated killing. This study presents pre-clinical evaluation of CVA21 using clinically relevant PDES samples and identifies CVA21 as a promising candidate for EWS immunotherapy.

INTRODUCTION

Ewing sarcoma (EWS) is a rare malignancy (90 cases/annum in the UK) of the bone and soft tissue, typically affecting young people aged between 10 and 25 years.[1] The 5-year survival for patients with localized disease is 50%–70%; however, for patients with metastatic and/or relapsed disease, this is reduced to only 20%–25%.[1] Unfortunately, 30% of patients with EWS have metastatic disease at diagnosis, and 25% of those with localized disease will relapse.[1] Current EWS treatments include a combination of multimodal chemotherapy, surgery, and/or radiotherapy. However, these treatments have limited efficacy against metastatic and/or relapsed disease[1] and are associated with significant morbidity (e.g., limb amputation, cardiopulmonary complications, and infertility).[2] Therefore, there is a desperate need for novel therapies that are less toxic to patients and more effective against metastatic and relapsed disease.

EWS is characterized by chromosomal translocations resulting in gene fusions between the *FET* and *ETS* gene families, most commonly (~85% of cases) between EWS breakpoint region-1 (*EWSR1*) and Friend leukemia integration-1 (*FLI1*), generating an aberrant transcription factor, EWSR1::FLI1,[3,4] which drives oncogenesis. Critically, fusion oncoprotein expression can vary within patient tumors, and within the same cells over time, creating a highly heterogeneous tumor.[5–7] The importance of the fusion oncoprotein in driving tumor progression is highlighted by the low mutational burden in EWS. Furthermore, this low mutational burden makes EWS weakly immunogenic.[8–10] However, despite EWS being immunologically cold and immunosuppressive,[11] immune cell infiltration by natural killer (NK) cells and/or CD8+ T cells is associated with improved survival.[12]

Unfortunately, EWS does not respond well to existing immunotherapies, such as immune checkpoint blockade.[13,14] Challenges for the success of immunotherapy in EWS include an immunosuppressive tumor microenvironment (TME), low immunogenicity,[8,15] minimal expression of classical immune checkpoints (e.g., programmed cell death ligand-1 [PD-L1][16]), and limited infiltration of anti-tumor immune cells.[17] Oncolytic viruses (OVs) are an emerging class of immunotherapy. The mechanisms of OVs are 2-fold: first, they cause direct cancer cell lysis (oncolysis) by preferentially replicating within cancer cells while sparing healthy cells. This releases progeny virus

Received 7 May 2026; accepted 13 October 2026;
https://doi.org/10.1016/j.omton.2026.201367.

Correspondence: Fiona Errington-Mais, Leeds Institute of Medical Research, University of Leeds, St James's University Hospital, Wellcome Trust Brenner Building, Beckett Street, Leeds LS9 7TF, UK.
E-mail: f.errington@leeds.ac.uk

Published by Elsevier Inc. on behalf of The American Society of Gene and Cell Therapy.
This is an open access article under the CC BY license (http://creativecommons.org/licenses/by/4.0/).

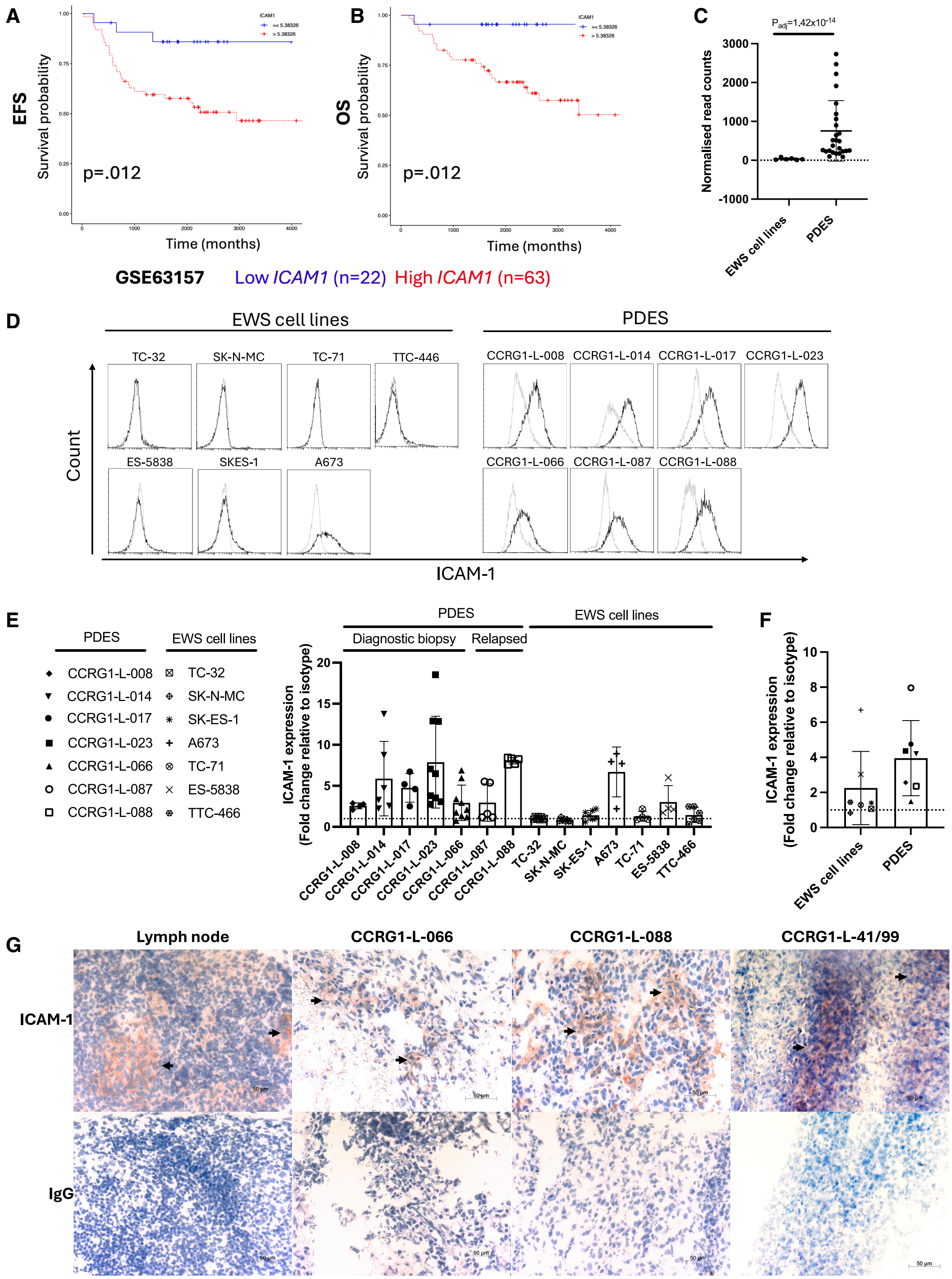

Figure 1. Expression of ICAM-1 in EWS

(A) Prognostic significance of *ICAM1* expression illustrated by Kaplan-Meier event-free survival (EFS) and (B) overall survival (OS) curves from a publicly available EWS dataset (GEO: GSE63157; $n = 85$; high *ICAM1* $n = 63$; low *ICAM1* $n = 22$). (C) Normalized read counts for *ICAM1* expression from bulk mRNA sequencing of EWS cell lines ($n = 6$) and PDES ($n = 26$). Data represents mean normalised read counts (±SD). Differential expression was identified using DESeq2.[35] (D) Representative flow cytometry histograms of

(legend continued on next page)

that can infect neighboring cancer cells, leading to further rounds of infection, replication, and oncolysis. Second, OVs activate both innate and adaptive anti-tumor immune responses. Specifically, OVs activate the immune system to (1) stimulate the production of pro-inflammatory/cytotoxic cytokines,[18] (2) recruit immune cells to generate an immunologically "hot" tumor,[18] (3) activate innate anti-tumor immunity via NK cells, and (4) prime adaptive (i.e., long-lasting) tumor-specific cytotoxic T lymphocytes (CTLs).[19] OVs have been trialed in various cancers to date, including in a handful of patients with EWS as part of broader pediatric cancer trials, and were safe and well-tolerated.[20–22]

Coxsackievirus A21 (CVA21; V937, formerly CAVATAK) is a wild-type enterovirus belonging to the *Picornaviridae* family with a single-stranded positive-sense RNA genome.[23] In immunocompetent adults, CVA21 typically causes mild upper respiratory tract infections.[24] The *Kyukendall* strain of CVA21 has been widely investigated as an OV that preferentially replicates in cancer cells due to overexpression of the viral entry receptor, intercellular adhesion molecule-1 (ICAM-1)/CD54; by contrast, most healthy tissues have low ICAM-1 expression.[23] CVA21 lyses cancer cells in an ICAM-1-dependent manner, stimulates NK cells to kill cancer cells, and primes adaptive T cell-mediated immune responses.[19,23] Upon clinical evaluation, CVA21 has demonstrated safety and efficacy in various cancers.[23,25–27]

This study aimed to test the efficacy of CVA21 against EWS and used patient-derived EWS cultures (PDES), recently isolated from patient tissues, including samples from diagnostic biopsy tissue and relapsed disease. These PDES have previously been characterized[28–31] and represent a population of mesenchymal-like, difficult-to-treat cancer cells found in many patient tumors; thus, these cells could contribute to disease relapse following primary treatment. Crucially, PDES are transcriptionally and phenotypically distinct from established EWS cell lines[31] and share similarities to matched patient tumors in terms of their mutational profile, transcriptomics, and response to chemotherapies and radiotherapy.[29,31] Unfortunately, no syngeneic EWS mouse models exist,[32] and patient-derived xenograft (PDX) models have a relatively poor (<25%) engraftment rate in immunocompromised mice,[33] which lack immune function to test CVA21 immunotherapy. Furthermore, CVA21 requires human ICAM-1,[23] highlighting the importance of human model systems, including PDES, to investigate the effectiveness of CVA21 against EWS. This study supports the use of CVA21 as a potential immunotherapy for EWS.

RESULTS

Expression of ICAM-1 in PDES and EWS cell lines

The expression of ICAM-1 has been previously associated with metastatic phenotypes in EWS cells and poor outcomes in patients.[34] We sought to investigate this further using publicly available datasets from patients with EWS. Indeed, microarray data (GEO: GSE63157) confirmed significantly worse event-free survival (EFS) and overall survival (OS) in patients with higher *ICAM1* expression (Figures 1A and 1B). These findings were supported by additional datasets (GEO: GSE17674 and GSE171618; Figures S1A and S1B), suggesting that *ICAM1* expression is associated with worse outcomes in patients with EWS.

The expression of ICAM-1 in EWS is heterogeneous and linked to the activity of the EWSR1::FLI1 fusion protein. For example, ICAM-1 is upregulated in EWS cells with low EWSR1::FLI1 fusion activity (EWSR1::FLI1^{low}) and downregulated in cells with high fusion activity (EWSR1::FLI1^{high}).[7,34,36–38] While highly proliferative EWS cell lines are EWSR1::FLI1^{high}, our unique panel of previously characterized PDES display a more mesenchymal-like phenotype, indicative of an EWSR1::FLI1^{low} state.[31] Given the inverse relationship between ICAM-1 expression and EWSR1::FLI1 activity, we hypothesized that ICAM-1 expression would be increased in PDES compared to the EWS cell lines.[37,38] Bulk mRNA sequencing confirmed this, showing that EWS cell lines (n = 6) have low *ICAM1* expression, while PDES (n = 26) express significantly higher levels (log2fold change = 4.2; adjusted p = 1.42e−14), albeit with a high degree of variability, reflecting interpatient heterogeneity (Figure 1C). Importantly, assessment of ICAM-1 surface protein expression on EWS cell lines and PDES using flow cytometry showed that five out of seven EWS cell lines lacked surface ICAM-1 (including A673 cells, in line with previously published findings[37–39]). By contrast, all PDES expressed ICAM-1 to varying degrees (Figures 1D and 1E), which was overall greater than observed in EWS cell lines (Figure 1F). To extend our findings to tumors, we subsequently examined the expression of ICAM-1 in EWS tissue by immunohistochemistry (IHC) (Figure 1G). As expected, this revealed heterogeneous ICAM-1 expression between patients but also within tumor sections. Therefore, based on the examined EWS tissue sections, it is likely that the EWSR1::FLI1^{low} cells exist as a minority population in tumors,[3] outnumbered by the more proliferative EWSR1::FLI1^{high} cells. Consistent with this, most cells within the samples lacked ICAM-1 expression; however, ICAM-1 staining was detected in six of eleven tumors, which was encouraging for a CVA21-focused strategy (Figures S1C and S1D; Table S1).

surface ICAM-1 expression (black line) on PDES and EWS cell lines compared to isotype control (gray line). (E) Relative surface ICAM-1 expression on PDES (n = 7) and EWS cell lines (n = 7); PDES and EWS cell lines have been allocated unique symbols, which are used in each figure. Data represent mean ICAM-1 expression (±SD) relative to isotype for $n \geq 3$ biological repeats. A dotted line at 1 has been added to illustrate the fold change above 1. (F) Direct comparison of mean relative ICAM-1 expression (±SD) in EWS cell lines (n = 7) and PDES (n = 7), as shown in (E). A dotted line at 1 has been added to illustrate the fold change above 1, and an unpaired t test was performed. (G) Representative examples of ICAM-1 staining in EWS tissue by IHC. Isotype control (IgG2b) was used as a negative control for each sample. Human lymph node tissue was used as a positive control, and a cytospin of ICAM-1-negative/low TC-32 cells was used as a negative control. Representative images are shown at 40× magnification. Scale bar: 50 μm. Black arrows indicate region of ICAM-1 staining.

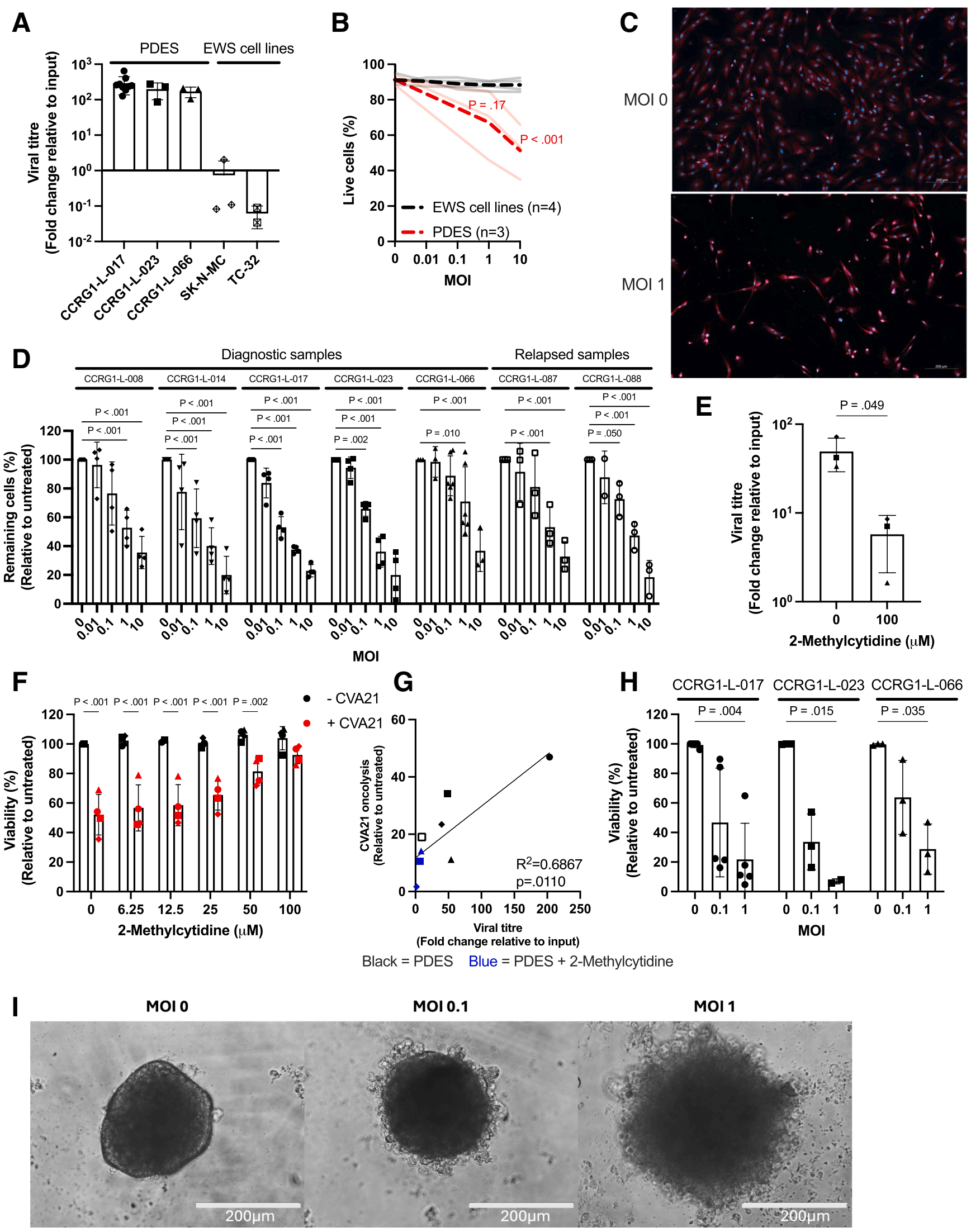

Figure 2. PDES are sensitive to CVA21 replication and oncolysis

(A) Mean fold change in CVA21 viral titer after PDES (n = 3) and EWS cell lines (n = 2) were infected with CVA21 (MOI 0.5). Total cell supernatant and lysates were collected 16 h post-infection, and CVA21 titers were measured via plaque assay. Data are presented as the mean fold change in viral titer compared to input virus for each cell culture (±SD). (B) Mean percentage of live EWS cell lines (n = 4; SK-N-MC, SKES-1, TC-32, and TTC-446) and PDES (n = 3; CCRG1-L-017, CCRG1-L-023, and CCRG1-L-066) after infection with CVA21 at increasing MOI for 48 h. Cell viability was measured using LIVE/DEAD flow cytometry. The bold dotted line represents mean cell viability for PDES (red) or EWS cell lines (black); faint solid lines represent individual PDES (red) or EWS cell lines (black). A two-way ANOVA with Šídák's multiple comparisons test was performed. (C) Representative images from Cell Discoverer 7 (CD7) high-content imaging. CCRG1-L-017 cells were infected with CVA21 (MOI 0 or 1; 48 h) before staining with DAPI (to identify nuclei; blue) and TOTO-3 (to identify cytoplasm; pink). Scale bar: 200 μm. (D) Percentage of remaining PDES from diagnostic biopsy (n = 5) and relapsed tissue (n = 2) following infection with increasing doses of CVA21 for 48 h. Live cells that remained adherent were stained as described in (C), and the percentage of remaining cells was measured using the CD7 high-content imager. Data are presented as the mean percentage of PDES remaining (±SD) compared to the untreated control. A two-way ANOVA with Dunnett's multiple comparisons test was performed. (E) Mean fold change in CVA21 viral titer after PDES (n = 3) were treated with or without 2′-C-methylcytidine (100 μM; 2 h) before infection with CVA21 (MOI 0.1). Total cell supernatant and lysates were collected 16 h post-infection, and CVA21 titers were measured via plaque assay. Data are presented as the mean fold change in viral titer compared to input virus for each treatment (±SD). A paired t test was performed. (F) Percentage viability of PDES (n = 4) following pre-treatment with or without increasing doses of 2′-C-methylcytidine (2 h) before infection with CVA21 (MOI 0.1). Cell viability was measured by CellTiter-Glo 48 h post-infection. Data are presented as the mean percentage viability of cells relative to the untreated control (±SD). A two-way ANOVA with Šídák's multiple comparisons test was performed. (G) Correlation analysis of relative CVA21 oncolysis (measured by CellTiter-Glo) versus viral replication (measured by plaque assay) of PDES (n = 5) and PDES pre-treated with 2′-C-methylcytidine (n = 3; 100 μM) 2 h prior to CVA21 treatment (MOI 0.1). A simple linear regression was performed. (H) Percentage viability of PDES (n = 3) 3D spheroids following infection with increasing doses of CVA21. Cell viability was measured by CellTiter-Glo 48 h post-infection. Data are presented as percentage viability relative to the untreated control (±SD). A two-way ANOVA with Šídák's multiple comparisons test was performed. (I) Representative bright-field images of PDES spheroids 48 h after treatment with CVA21. Scale bar: 200 μm.

PDES expressing ICAM-1 are sensitive to CVA21 infection and oncolysis

CVA21 can infect cancer cells via its entry receptor ICAM-1, resulting in viral replication and oncolysis. We therefore examined whether PDES and EWS cell lines could support CVA21 replication. PDES (n = 3) and EWS cell lines (n = 2) were infected with CVA21 (multiplicity of infection [MOI] 0.5) for 16 h before cell lysates were collected and viral replication was measured by plaque assay. All PDES tested displayed an approximately 100-fold increase in viral output compared to input titers, while CVA21 failed to replicate in ICAM-1-negative EWS cell lines (Figure 2A). CVA21 oncolysis was then assessed by treating PDES and EWS cell lines with increasing MOIs of CVA21 for 48 h before measuring cell viability by flow cytometry. Supporting the viral replication data, EWS cell lines were insensitive to CVA21, while PDES were killed following CVA21 infection (Figure 2B).

We further assessed the oncolytic ability of CVA21 and expanded our study to a wider panel of PDES. Due to the slow doubling time of some of the PDES,[31] oncolysis was quantified using high-content imaging, which required fewer cells than flow cytometry (Figure 2C). All PDES tested were highly sensitive to CVA21 oncolysis 48 h post-infection, in a dose-dependent manner (Figure 2D). Encouragingly, CVA21 was effective at lysing PDES derived from both diagnostic biopsy tissues and relapsed samples. Pre-treatment with 2′-C-methylcytidine (a nucleoside analog that inhibits viral/CVA21 replication; Figure 2E) prior to CVA21 treatment inhibited oncolysis in a dose-dependent manner (Figure 2F), indicating that CVA21-induced oncolysis was replication dependent. Further supporting the importance of viral replication, a significant positive correlation ($R^2 = 0.6867$; $p = 0.011$) was observed between CVA21 oncolysis and viral replication (Figure 2G). Finally, PDES ($n = 3$) were grown as 3D tumor spheroids before CVA21 treatment and remained sensitive to CVA21 oncolysis (Figure 2H), demonstrating CVA21-mediated disruption of spheroid integrity (Figure 2I). Interestingly, PDES 3D spheroid cultures appeared more sensitive to CVA21 oncolysis than in 2D monolayer cultures (e.g., at MOI 1, the mean cell viability for CCRG1-L-066 spheroids was 29%, while in 2D, cell viability was only reduced to 71%); enhanced viral spread is likely due to the close proximity of cells within the spheroid. Together, these results demonstrate that PDES express sufficient ICAM-1 on their surface to support CVA21 infection, replication, and oncolysis, while established EWS cell lines do not.

CVA21 induces innate immune responses toward EWS cells

Although traditionally considered to be an immunologically “cold” tumor, EWS tissue was recently reported to be more infiltrated with immune cells than previously thought,[40] containing cell populations including NK cells, T cells, dendritic cells, and immunosuppressive macrophages. Based on these observations, we adjusted our modeling of the TME to explore the role of immune cells (healthy donor peripheral blood mononuclear cells [PBMCs]) on OV efficacy in PDES. PBMCs contain relevant immune cell subsets identified in EWS,[6,40] although they have not undergone pre-conditioning within the TME. Importantly, PBMCs have the capacity to induce pro-inflammatory and antiviral cytokines in response to OVs, which could ultimately influence OV efficacy.[18]

Immune activation and immune-mediated cytotoxicity are critical modes of action for OVs, including CVA21. We therefore chose to examine the ability of immune cells to kill PDES in the presence or absence of CVA21. Firstly, we investigated whether CVA21-stimulated PBMCs could kill uninfected PDES. To do this, PBMCs were stimulated with CVA21 for 48 h prior to a short-term (5 h) co-culture in suspension with uninfected SK-N-MC, TC-32, and PDES; cell viability was then determined by flow cytometry. These data demonstrated a significant reduction in SK-N-MC, TC-32, and PDES viability ($n = 3$ PDES; Figures 3A and 3B, respectively) upon CVA21 stimulation and suggest that CVA21-stimulated PBMCs can kill EWS cells, irrespective of ICAM-1 expression. No direct oncolytic effects of CVA21 were observed in PDES following 5 h of CVA21 treatment (Figure S2).

Next, we modeled a scenario in which SK-N-MC, TC-32, and PDES were treated with a low dose of CVA21 prior to co-culture with PBMCs with no prior activation by CVA21, to mimic the initial stages of CVA21 infection and immune influx in the TME. Adherent SK-N-MC, TC-32, and PDES ($n = 5$) were infected with an intermediate dose of CVA21 (MOI 0.5) for 2 h prior to co-culture with unstimulated PBMCs for a further 48 h, and EWS cell viability was measured by flow cytometry. As expected, SK-N-MC and TC-32 cell line viability was not reduced following CVA21 infection in the absence of PBMCs, while co-culture with PBMCs in the absence of CVA21 infection decreased EWS cell line viability, which was further reduced following CVA21 stimulation (Figure 3C); these data are consistent with our previous findings using an alternative OV (Maraba virus; MG1).[41] By contrast, PDES viability was significantly decreased following CVA21 infection in the absence of PBMCs, demonstrating direct oncolysis for four of five PDES, while PBMCs alone caused a mild but significant decrease in PDES viability for three of five PDES (Figure 3D); crucially, PDES viability was significantly decreased (compared to untreated, CVA21 alone, or PBMCs alone) for all five PDES when PBMCs were co-cultured with CVA21-infected cells (Figure 3D). To explore the potential role for direct oncolysis or immune-mediated killing in PDES, plaque assays were performed using one PDES (CCRG1-L-017) in the presence or absence of different PBMC donors ($n = 7$). These data revealed a significant decrease in CVA21 viral replication in the presence of PBMCs (Figure 3E), indicating that decreased PDES viability in CVA21-treated co-cultures was most likely due to immune-mediated killing rather than enhanced CVA21 replication/oncolysis.

Critically, the SK-N-MC and TC-32 cell lines used herein lack sufficient ICAM-1 expression (Figure 1E) to support CVA21 infection/oncolysis (Figures 2A and 2B) but displayed a significant decrease in cell viability when co-cultured with CVA21-stimulated PBMCs, consistent with the ability of CVA21 to activate immune-mediated killing and previous findings.[19,42] Using this non-permissive CVA21 model system, we then investigated the potential mechanisms responsible for the immune-mediated killing, focusing on the importance of NK cells and/or CVA21-induced cytokines in a setting where direct oncolysis was absent. Firstly, to explore the role of CVA21-induced cytokines, conditioned media from PBMCs that had been left unstimulated (PBMC-CMs) or stimulated with CVA21 for 48 h (CVA21-stim PBMC-CMs) were used to assess EWS cell line viability. CVA21-stim PBMC-CM caused a small (6%) but significant decrease in the viability of SK-N-MC cells; this reduction was not observed for TC-32 cells, although there was more variation in cytotoxicity between PBMC donors for this cell line (Figure 4A). Similar findings were also observed using PDES (Figure S3A). Therefore, the lack of cytotoxicity observed from CVA21-stim PBMC-CM treatment suggested an alternative mechanism of action. Next,

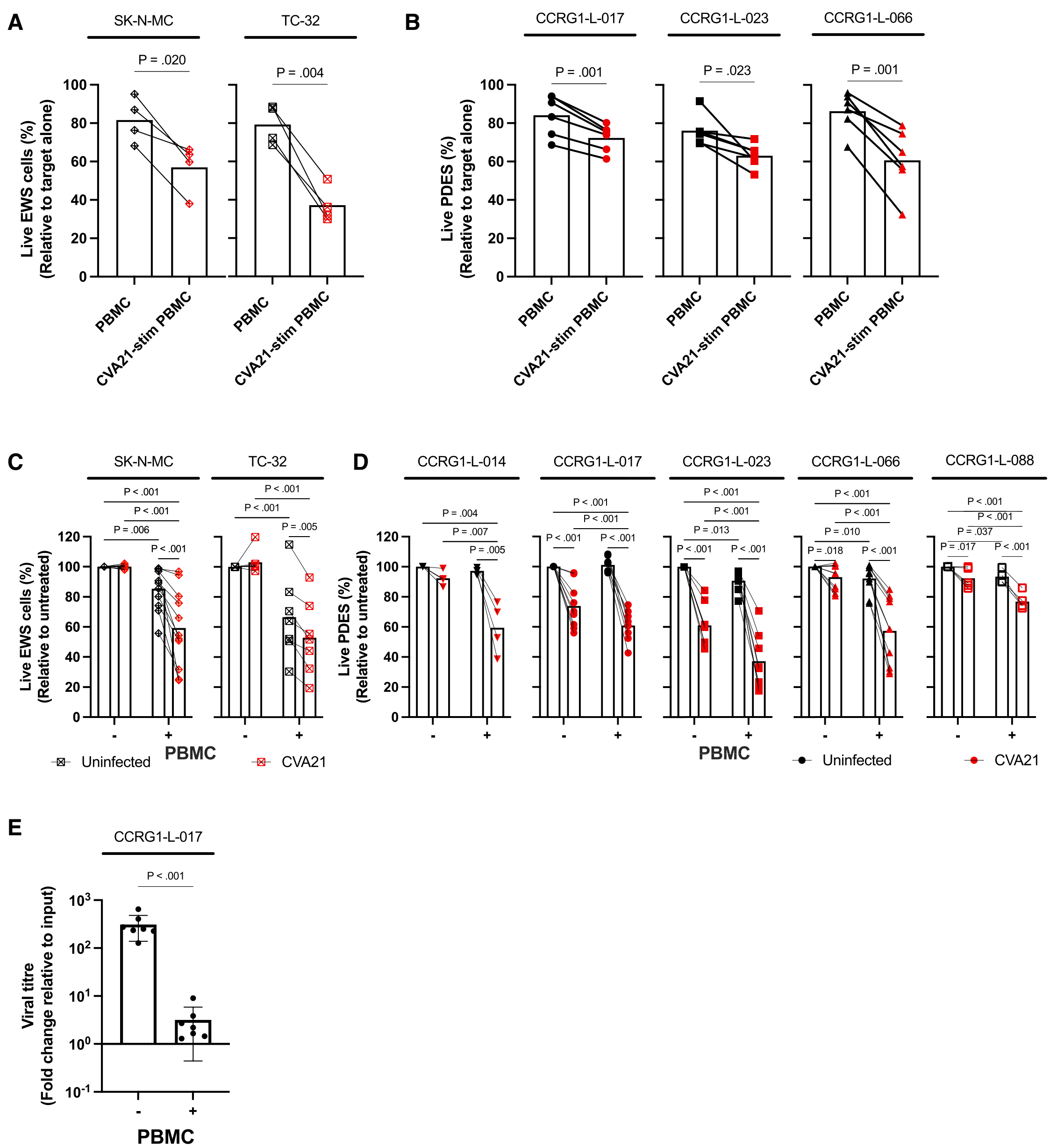

Figure 3. CVA21 stimulates immune-mediated killing of EWS cells

(A) Percentage of live EWS cell lines (*n* = 2) and (B) PDES (*n* = 3) after short-term suspension co-culture (25:1 effector:target ratio; 5 h) with unstimulated or CVA21-stimulated PBMCs (MOI 0.1; 48 h). *n* = 4–6 PBMC donors for each experiment. Cell viability was measured using LIVE/DEAD flow cytometry, and data are presented as the mean percentage of live target cells ($CD45^{-}$) relative to target cells in the absence of PBMCs. Each line represents a different PBMC donor. A paired *t* test was performed for each EWS cell line or PDES. (C) Percentage of live EWS cells and (D) PDES following CVA21 infection (MOI 0.5) and longer-term adherent co-culture with PBMCs (10:1 effector:target ratio; 48 h). *n* = 2 EWS cell lines and *n* = 5 PDES; $n \geq 3$ PBMC donors for each EWS cell line or PDES. Cell viability was measured using LIVE/DEAD flow cytometry, and data are presented as the mean percentage of live target cells ($CD45^{-}$) relative to untreated target cells in the absence of PBMCs; each line represents a different PBMC donor. A two-way ANOVA with Fisher's post hoc test was performed. (E) Fold change in CVA21 viral titer after PDES (*n* = 1) were infected with CVA21 (MOI 0.1) for 2 h before co-culture with or without PBMCs (*n* = 7 donors) for 16 h. Total cell lysates were harvested, and viral titer was measured by plaque assay. Data are presented as the mean fold change in viral titer compared to input virus (±SD).

the role of NK cells was explored by depleting NK cells from PBMCs immediately prior to co-culture with EWS cells; NK cell depletion was confirmed (Figure S3B). Using both the 5-h short-term killing assay (with prior CVA21 stimulation of PBMCs) and the 48-h killing assay (with simultaneous CVA21/PBMC treatment), we confirmed significant immune-mediated

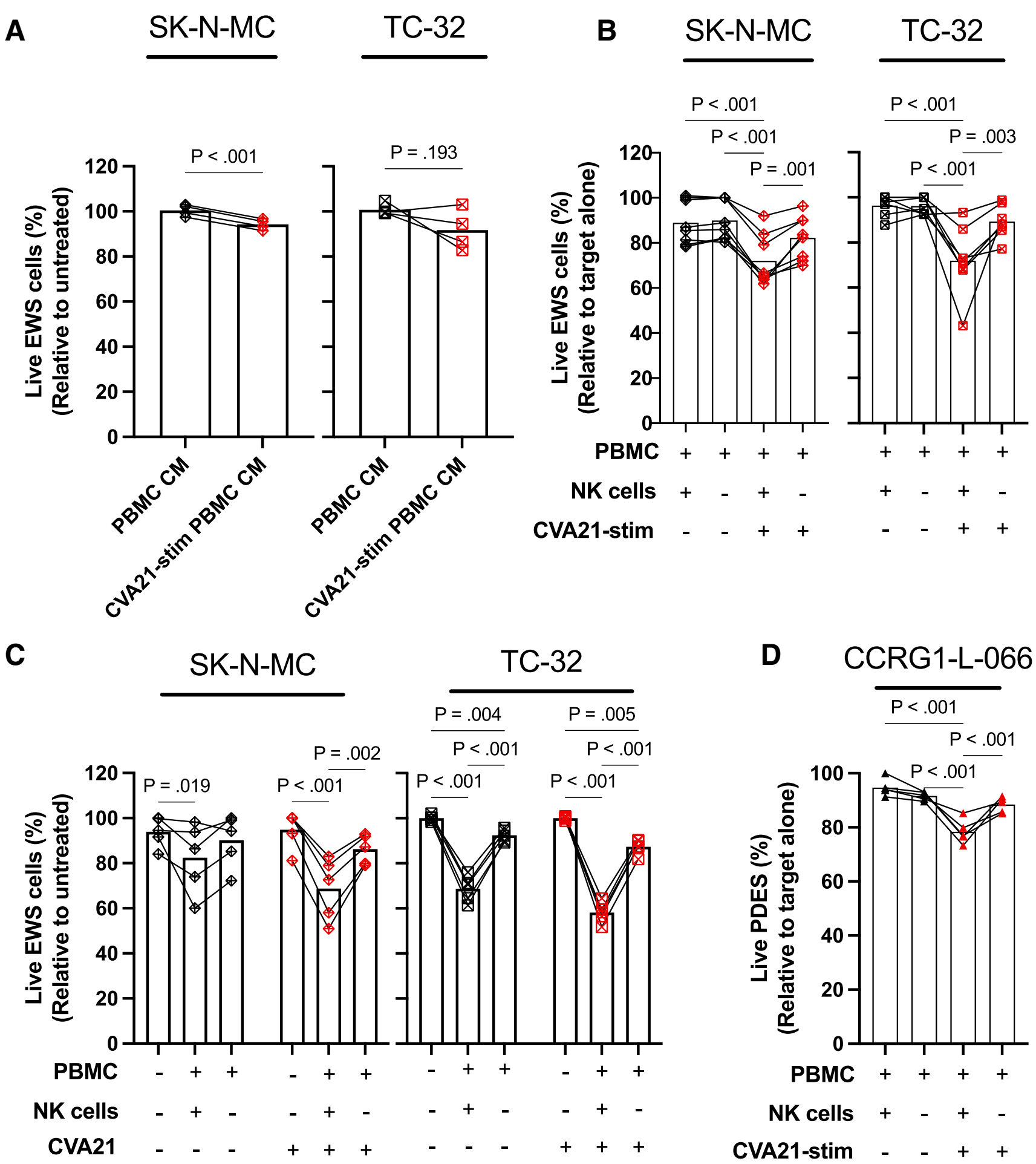

Figure 4. NK cells play a key role in CVA21-stimulated immune-mediated cytotoxicity of EWS cells

(A) Percentage of live EWS cell lines (n = 2) after 48 h treatment with PBMC-CM or CVA21-stimulated CM (MOI 0.1; 48 h). Cell viability was measured using LIVE/DEAD flow cytometry, and data are presented as the mean percentage of live EWS cells relative to untreated cells. A paired t test was performed. (B) Percentage of live EWS cell lines after short-term suspension co-culture (25:1 effector:target ratio; 5 h) with unstimulated/CVA21-stimulated whole PBMCs or NK cell-depleted PBMCs (MOI 0.1; 48 h). n = 7–8 PBMC donors for each. Cell viability was measured using LIVE/DEAD flow cytometry, and data are presented as the mean percentage of live target cells ($CD45^-$) relative to untreated target cells in the absence of PBMCs. Each line represents a different PBMC donor. A paired t test was performed for each EWS cell line. (C) Percentage of live EWS cell lines after CVA21 infection (MOI 0.5) and longer-term adherent co-culture with PBMCs or NK cell-depleted PBMCs (10:1 effector:target ratio; 48 h). n = 2 EWS cell lines. n = 5 PBMC donors for each EWS cell line. Cell viability was measured using LIVE/DEAD flow cytometry, and data are presented as the mean percentage of live EWS cells ($CD45^-$) relative to untreated target cells in the absence of PBMCs; each line represents a different PBMC donor. A two-way ANOVA with Fisher's post hoc test was performed. (D) Experiment as described in (A) using PDES (n = 1). A paired t test was performed

killing when CVA21-treated whole PBMCs were used; by contrast, significant immune-mediated killing was not observed when using NK cell-depleted PBMCs, irrespective of CVA21 activation (Figures 4B and 4C). Finally, to confirm the importance of NK cells in the immune-mediated killing of PDES (Figure 3B), the 5-h co-culture assay was used to exclude direct effects of CVA21 oncolysis; these data confirmed the role of NK cells, with PBMC-mediated killing reduced in the absence of NK cells (Figure 4D). Although the results are more difficult to interpret in the 48-h co-culture assay due to CVA21 oncolysis, PDES viability was significantly decreased (compared to CVA21 alone) when whole PBMCs were co-cultured with CVA21-infected cells; however, the significance of this was diminished when NK cells were removed (Figure S3C).

Together, these findings indicate that CVA21 can stimulate NK cell-mediated cytotoxicity toward EWS cells, even when cells lack ICAM-1 expression and are insensitive to direct CVA21 oncolysis.

CVA21 replication and oncolysis are inhibited by type I interferon

OVs are known to stimulate a pro-inflammatory antiviral immune reaction. Indeed, we have previously demonstrated that PBMCs secrete a host of pro-inflammatory and antiviral cytokines in response to CVA21, including type I interferon α (IFNα)[19,43]; these cytokines are critical for the generation of CVA21-mediated innate and adaptive anti-tumor immune responses.[19]

Given the observed decrease in CVA21 replication when PDES were co-cultured with PBMCs (Figure 3E), we sought to investigate the effects of recombinant IFNα on CVA21 replication and oncolysis. Pre-treatment with IFNα effectively inhibited CVA21 replication at MOI 0.1 and 0.5 (Figure 5A) and caused a significant reduction in oncolysis (Figure 5B). However, PDES were incapable of producing type I IFN themselves when infected with CVA21 (Figures S4A and S4B). These findings suggest that PDES would respond to exogenous IFNα, produced by CVA21-stimulated immune cells within the TME, thus triggering an antiviral state that inhibits CVA21 replication and oncolysis. To investigate this further, we examined whether recombinant IFNα and PBMC-CM collected after CVA21 stimulation could induce phosphorylation of STAT1, a critical mediator of the cellular response to IFN and the control of viral infection.[44] STAT1 phosphorylation was clearly evident in two of three PDES following IFNα treatment (Figures 5C and 5D) and CVA21-stimulated PBMC-CM (Figure 5E), suggesting heterogeneity in the response. However, these data

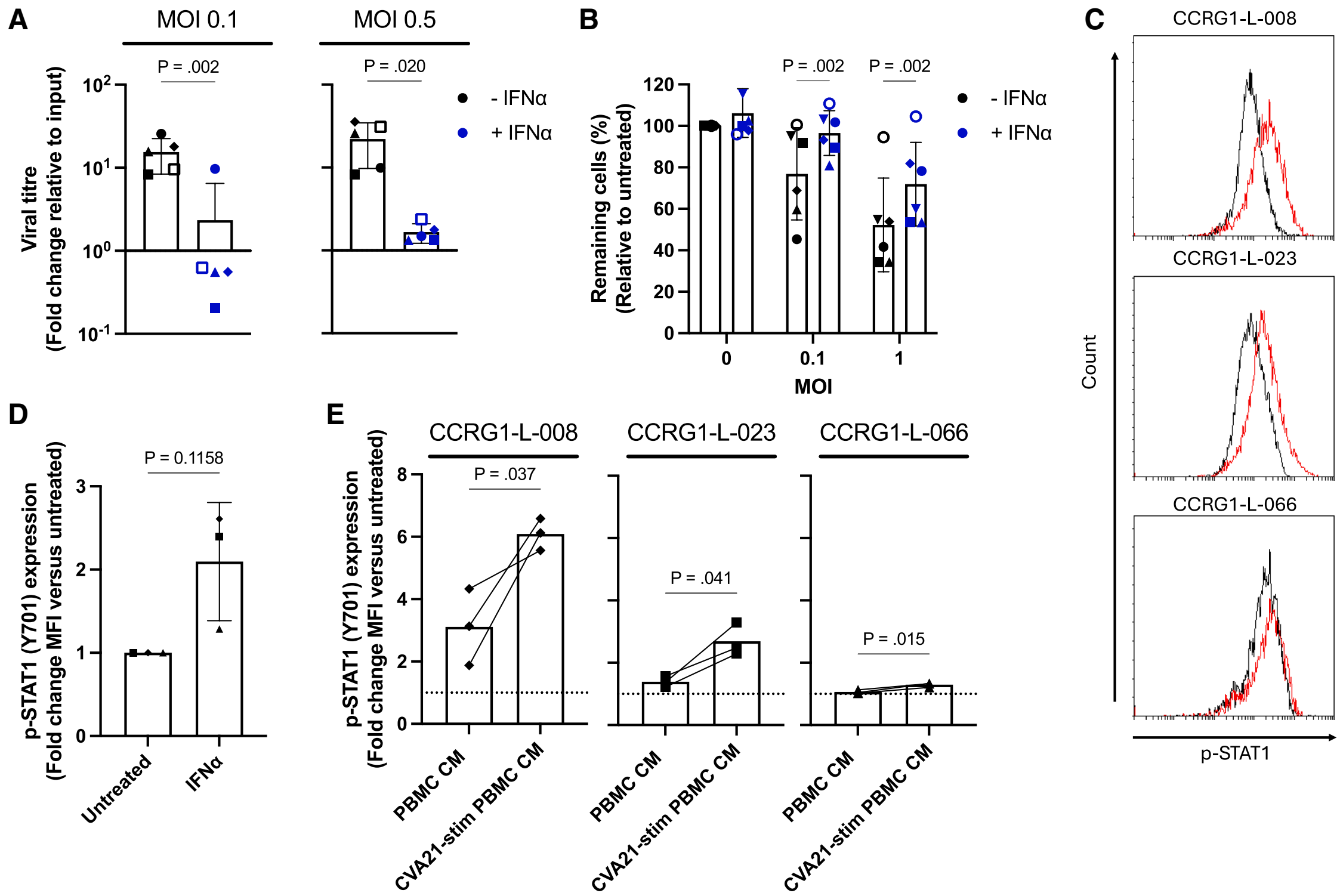

Figure 5. IFNα stimulates antiviral signaling, inhibiting CVA21 replication and oncolysis

(A) Fold change in CVA21 viral titer after PDES (n = 5) were pre-treated with or without IFNα (5 ng/mL; 24 h) before infection with CVA21 (MOI 0.1 and 0.5). Total cell supernatant and lysates were collected 16 h post-infection, and viral titers were measured via plaque assay. Data are presented as the mean fold change in viral titer compared to input virus for each PDES (±SD). A paired t test was performed. (B) Percentage viability of PDES (n = 5) following pre-treatment with or without IFNα (5 ng/mL; 24 h) before infection with increasing doses of CVA21. Cell viability was measured by CellTiter-Glo 48 h post-infection. Data are presented as the mean percentage viability (±SD) relative to the untreated control. A two-way ANOVA with Šídák's multiple comparisons test was performed. (C) Representative flow cytometry histograms of intracellular phospho-STAT1 (pSTAT-1; Y701) on untreated (black lines) and IFNα-treated (red lines; 5 ng/mL; 30 min) and (D) relative pSTAT-1 expression on PDES (n = 3) treated with or without IFNα as described in (C). Data represent the mean fold change of MFI relative to untreated (±SD). A paired t test was performed. (E) Relative pSTAT-1 expression on PDES (n = 3) treated with PBMC-CM or CVA21-stimulated (MOI 0.1; 48 h) PBMC-CM (1:1 RPMI:CM ratio; 30 min). Data represent the mean fold change of MFI relative to untreated for each PDES (±SD). Paired t tests were performed.

demonstrated the ability of PDES to respond to type I IFNα to varying levels and that exposure to CVA21-induced cytokines also triggered a similar response.

Increasing ICAM-1 expression to enhance oncolysis

Given the strong relationship between ICAM-1 expression and CVA21 sensitivity,[19,42] we hypothesized that a further increase in ICAM-1 expression may sensitize PDES to CVA21 oncolysis even in the presence of antiviral immune responses, thus switching the balance in favor of viral replication/oncolysis. As a proof-of-principle experiment, we used the pro-inflammatory cytokine, tumor necrosis factor (TNF), to upregulate ICAM-1. Pre-treatment of n = 7 PDES with TNF for 24 h increased surface ICAM-1 expression in all PDES (Figures 6A and 6B). This TNF-mediated increase in ICAM-1 expression also enhanced CVA21 viral replication across each PDES tested (Figure S5), although this observation was dependent on the MOI used for each PDES. Crucially, increased ICAM-1 expression sensitized PDES to CVA21 oncolysis (Figure 6C), both in PDES from diagnostic biopsies (n = 5) and relapsed tissue (n = 2). Increasing ICAM-1 expression with TNF also conferred a significant enhancement of oncolysis in 3D spheroid cultures at lower MOIs (Figure 6D).

Next, EWS cell lines lacking ICAM-1 expression were used to investigate whether ICAM-1-negative cells could be sensitized to CVA21 oncolysis by TNF treatment. Notably, three EWS cell lines exhibited a subpopulation of cells that upregulated ICAM-1 expression following pre-treatment with TNF (termed responders; ES-5838, SK-ES-1, and A673, Figures 6E and 6F). However, other EWS cell lines did not upregulate ICAM-1 expression in response to TNF (termed non-responders; TC-32, SK-N-MC, TC-71, and TTC-446, Figures 6E and 6F). Accordingly, the TNF responder group of EWS cell lines became significantly sensitized to CVA21 oncolysis, while the non-responder group remained insensitive (Figure 6G).

TNF can, in some instances, directly cause cell death. Therefore, we sought to address whether the enhancement of cytotoxicity observed

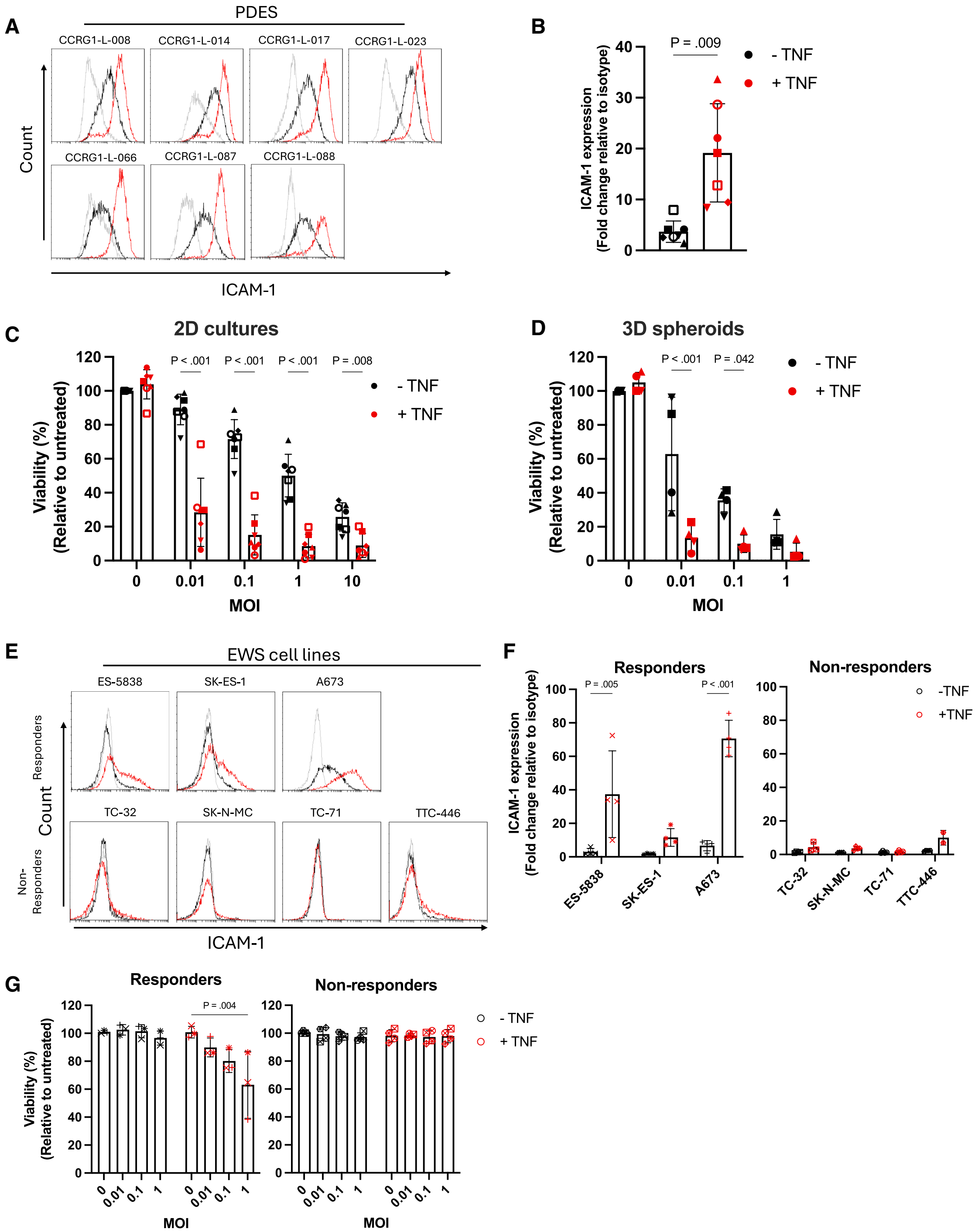

Figure 6. TNF pre-treatment increases ICAM-1 expression and enhances CVA21 oncolysis

(A) Representative flow cytometry histograms of PDES surface ICAM-1 expression on untreated (black line) and TNF-treated (5 ng/mL; 24 h; red line) cells compared to isotype control (gray line). (B) Relative surface ICAM-1 expression on PDES (n = 7) after treatment described in (A). ICAM-1 expression was measured by flow cytometry, and data represent the mean fold change in ICAM-1 expression relative to the isotype control (±SD). A paired t test was performed. (C) Percentage cell viability of PDES (n = 7) in 2D culture and (D) 3D spheroids following pre-treatment with or without TNF (5 ng/mL; 24 h) before infection with increasing doses of CVA21. Cell viability was measured by CellTiter-Glo 48 h post-infection. Data are presented as the mean percentage viability of cells relative to untreated controls (±SD). A two-way ANOVA with Šídák's multiple comparisons test was performed. (E) Representative flow cytometry histograms of EWS cell line surface ICAM-1 expression on untreated (black line) and

(legend continued on next page)

in Figure 6C was indeed due to increased CVA21 replication/oncolysis rather than cytokine-mediated apoptosis (i.e., was CVA21 infection sensitizing PDES to TNF-mediated apoptosis or was TNF sensitizing PDES to CVA21 infection and oncolysis). Accordingly, PDES were pre-treated with the viral replication inhibitor 2′-C-methylcytidine (100 μM) prior to infection with CVA21 (MOI 0.1), followed by treatment with TNF 24 h later (to ensure equal levels of ICAM-1 expression and viral entry). 2′-C-methylcytidine effectively restored viability both in the presence and absence of TNF (Figure 7A), confirming that the increased cytotoxicity was indeed dependent on viral replication/oncolysis and not viral sensitization to TNF-mediated cytotoxicity.

Based on the observation that increasing ICAM-1 expression can enhance CVA21 oncolysis, we assessed the correlation between relative ICAM-1 expression and relative sensitivity to CVA21 oncolysis in EWS cell lines (low ICAM-1 expression), PDES (moderate ICAM-1 expression), and PDES treated with TNF (high ICAM-1 expression) (Figure 7B). These data revealed a significant positive correlation ($R^2 = 0.7241$; $p < 0.0001$ and 0.6280; $p < 0.0001$ at MOI 0.1 and 1, respectively) between ICAM-1 expression and sensitivity to CVA21 oncolysis.

Given that increasing ICAM-1 expression could enhance CVA21 oncolysis, we investigated whether increasing ICAM-1 expression could restore oncolysis in the presence of antiviral type I IFNα (Figure 5). PDES (n = 6) were pre-treated with recombinant TNF, IFNα, or a combination of both prior to infection with CVA21. To observe enhancement or inhibition of CVA21-mediated killing, an intermediate dose of CVA21 was used (MOI 0.1). CVA21 infection alone reduced viability to ∼77%, with TNF treatment further reducing cell viability to ∼39%. As expected, IFNα treatment significantly inhibited oncolysis and restored viability to ∼96%. Encouragingly, when PDES were pre-treated with both TNF and IFNα, oncolysis was restored to levels equivalent to CVA21 alone (∼70%) (Figure 7C). These data suggest that TNF increased ICAM-1 expression and enhanced CVA21 infection could counteract the inhibitory effects of IFNα. This is encouraging, as both TNF and IFNα are released by immune cells following CVA21 treatment and are important mediators of OV-induced anti-tumor immune responses.[19] Moreover, these findings support the ability of CVA21 to maintain its oncolytic activity while stimulating the immune system.

Increasing ICAM-1 expression and CVA21 oncolysis using all-*trans* retinoic acid

As demonstrated in Figure 7, sensitivity to CVA21 oncolysis is strongly correlated with ICAM-1 expression on PDES, making ICAM-1 a candidate biomarker of response to CVA21. However, TNF is not suitable for direct administration to patients; therefore, we sought to identify a clinically relevant means of increasing ICAM-1 expression to enhance CVA21 oncolysis in the context of an antiviral immune response. All-*trans* retinoic acid (ATRA) is a safe and clinically relevant drug that is commonly used to treat skin conditions, including acne,[45] and hematological malignancies, such as acute promyelocytic leukemia.[46] ATRA has also been reported to increase ICAM-1 expression.[47,48] We initially treated PDES (n = 5) with increasing concentrations of ATRA for 72 h and observed a significant dose-dependent increase in ICAM-1 expression (Figure 8A). Following these observations, we pre-treated PDES (n = 5) with 1 μM ATRA for 72 h (Figure 8B) before treatment with CVA21. Similarly to TNF pre-treatment, ATRA pre-treatment also caused a significant increase in CVA21-mediated oncolysis (Figure 8C). Furthermore, ICAM-1 expression was also increased in the TNF/ICAM-1-responding EWS cell lines (Figures 6E–6G) following ATRA pre-treatment (Figures S6A and S6B) and were sensitized to CVA21 oncolysis (Figure S6C). By contrast, the TNF/ICAM-1-non-responding EWS cell lines did not respond to ATRA pre-treatment and accordingly were not sensitized to CVA21 oncolysis. Importantly, these data mirror those obtained with TNF (Figures 6E–6G) and suggest that some ICAM-1-low/negative EWS cells can be sensitized to CVA21 direct oncolysis, but others cannot.

As ATRA can induce cell differentiation and inhibit proliferation,[46,49] we next confirmed that ATRA-dependent enhancement of cytotoxicity was due to increased CVA21 oncolysis rather than cytostatic effects, which could be misrepresented as CVA21-induced cytotoxicity using the CellTiter-Glo assay (Figure S7). To do this, we repeated the same experiment but also quantified the percentage of viable/dead PDES (n = 5) in the total cell population using Live/Dead flow cytometry. These data confirmed that ATRA alone did not cause direct cytotoxicity; by contrast, pre-treatment with ATRA caused a significant reduction in the number of live cells following CVA21 infection (Figure 8D). Furthermore, pre-treatment of PDES spheroids (n = 5) with ATRA also significantly enhanced CVA21 oncolysis at MOI 0.01, although this increase was not significantly increased at higher MOIs (Figure 8E).

We then explored whether ATRA pre-treatment could simulate the effects of TNF and potentially counteract antiviral IFNα effects, as observed in Figure 7C. Again, an intermediate dose of CVA21 was used (MOI 0.1) to allow detection of enhanced or inhibited CVA21-mediated killing. Encouragingly, ATRA was able to restore CVA21 oncolysis in the presence of recombinant IFNα in n = 3/5

TNFα-treated (5 ng/mL; 24 h; red line) cells compared to isotype control (gray line). (F) Relative surface ICAM-1 expression on EWS cell lines (n = 7) after treatment described in (E). EWS cell lines were grouped as responders (ES-5838, SK-ES-1, and A673) and non-responders (TC-32, SK-N-MC, TC-71, and TTC-446). A two-way ANOVA with Šídák's multiple comparisons test was performed. (G) Percentage cell viability of EWS cell lines (n = 7) following pre-treatment with or without TNF (5 ng/mL; 24 h) before infection with increasing doses of CVA21. Cell viability was measured by CellTiter-Glo 48 h post-infection. Data show the mean percentage viability of cells relative to the untreated control (±SD); EWS cell lines were grouped as responders and non-responders in terms of ICAM-1 upregulation in (A). A Two-way ANOVA with Dunnett's multiple comparisons test was performed.

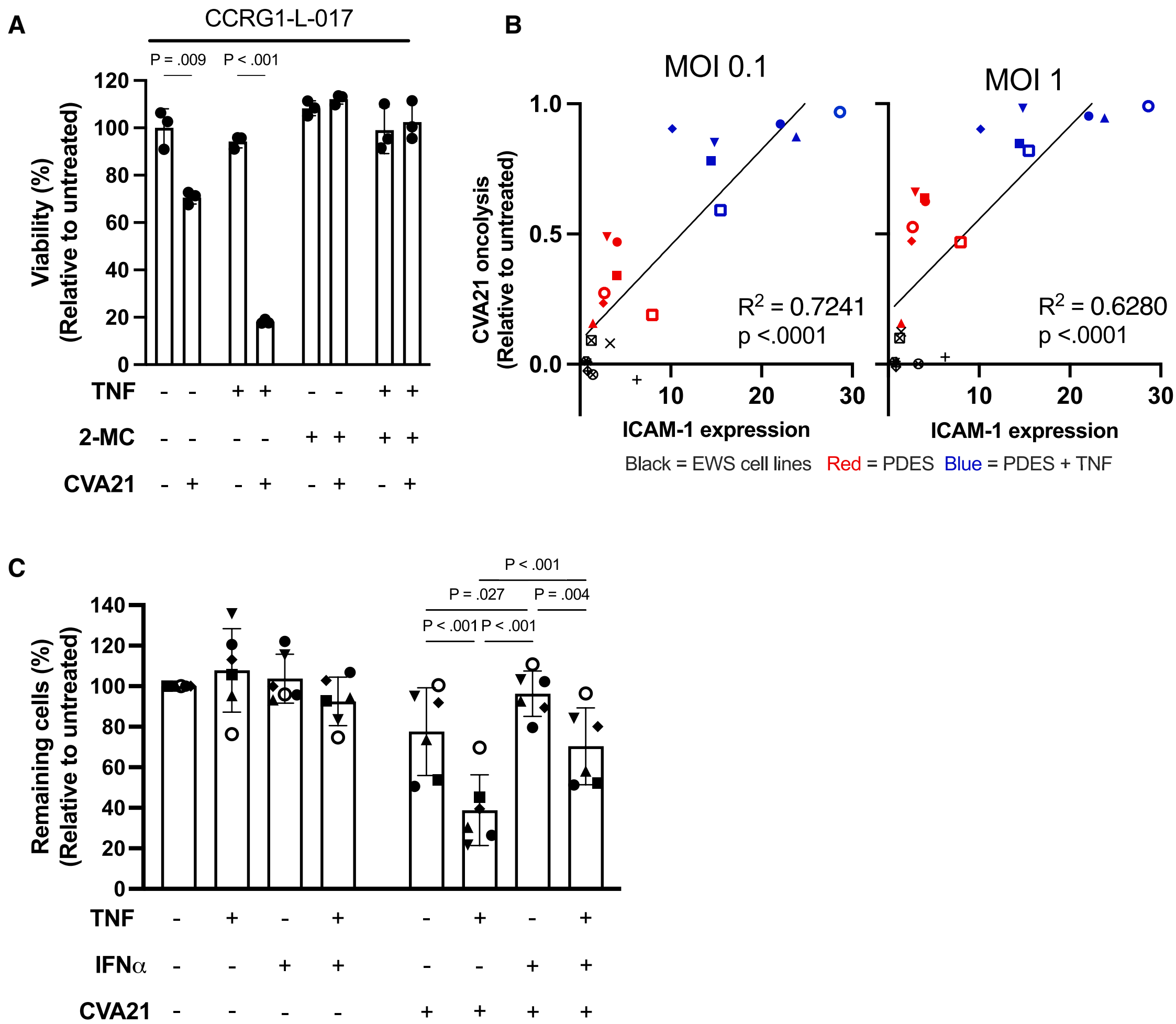

Figure 7. TNF-induced increase in ICAM-1 expression boosts CVA21 oncolysis and counterbalances antiviral IFNα

(A) Percentage cell viability of PDES (*n* = 1) following co-treatment of 2′-C-methylcytidine (2-MC; 100 μM) and CVA21 (MOI 0.1) for 24 h before further administration of TNF (5 ng/mL; 24 h). Cell viability was measured by CellTiter-Glo 48 h post-infection. Data are presented as the mean percentage viability of cells relative to untreated controls (±SD). A two-way ANOVA with Šídák's multiple comparisons test was performed. (B) Correlation analysis of relative CVA21 oncolysis (measured by CellTiter-Glo) versus relative ICAM-1 expression (measured by flow cytometry) of EWS cell lines (*n* = 7), PDES (*n* = 7), and PDES pre-treated with TNF (5 ng/mL; 24 h) (*n* = 7) at MOI 0.1 and 0.5. A simple linear regression was performed. (C) Percentage cell viability of PDES (*n* = 6) following pre-treatment with IFNα (5 ng/mL; 24 h) or TNF (5 ng/mL; 24 h) alone or in combination, before infection with CVA21 (MOI 0.1). Cell viability was measured by CellTiter-Glo 48 h post-infection. Data are presented as the mean percentage viability relative to the untreated controls (±SD). A two-way ANOVA with Fisher's multiple comparisons test was performed.

PDES (Figure 8F), suggesting that ATRA could be used clinically in some patients with EWS to increase ICAM-1 expression prior to CVA21 treatment.

Finally, we investigated whether the increase in ICAM-1 expression induced by ATRA could counteract inhibitory antiviral signaling induced by CVA21-stimulated PBMCs. To investigate this, PDES were pre-treated with 1 μM ATRA for 72 h to increase ICAM-1 expression. Medium was then replaced with PBMC-CM collected from unstimulated (IFNα-low) or CVA21-stimulated (IFNα-high) PBMCs,[19] before infecting PDES with CVA21. ATRA pre-treatment significantly increased oncolysis in the presence of both unstimulated and CVA21-treated PBMC-CMs (Figure 8G), thus restoring sensitivity in the presence of antiviral IFN. Together, these findings suggest that ATRA could be used to increase ICAM-1 expression on PDES and enhance CVA21 oncolysis in the presence of antiviral immune signaling. These findings provide a potential therapeutic approach for EWS using ATRA as an effective combination partner to boost CVA21 efficacy.

DISCUSSION

Patients with EWS need kinder and more effective treatments, particularly those with relapsed/refractory and metastatic disease. ICAM-1 has been identified as a significant risk factor in patients with EWS, with higher levels of ICAM-1 associated with metastatic disease and reduced survival.[34] Our study confirms this finding

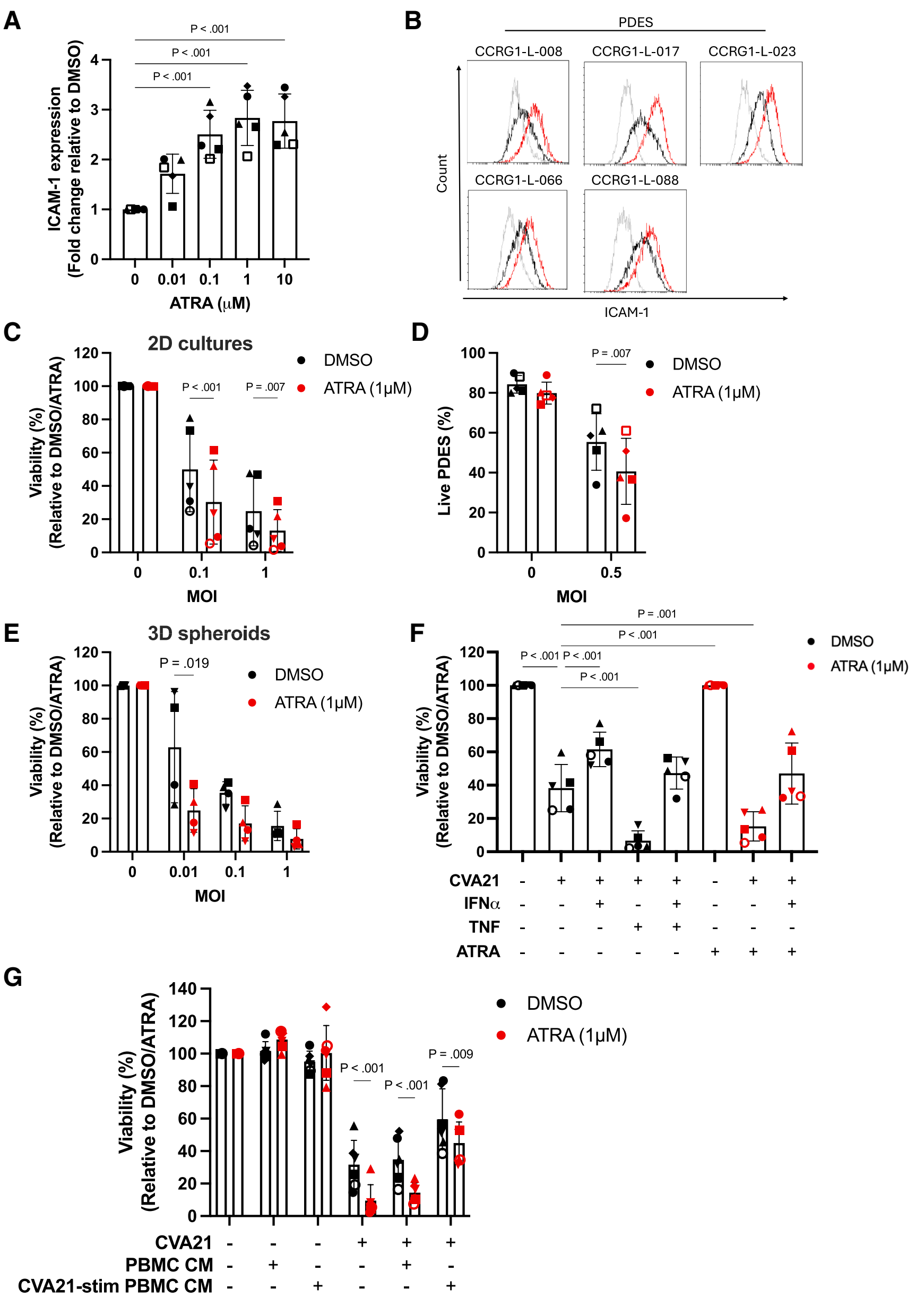

Figure 8. All-*trans* retinoic acid pre-treatment increases ICAM-1 expression and enhances CVA21 oncolysis of PDES

(A) Surface ICAM-1 expression on PDES (n = 5) following treatment with increasing concentrations of all-*trans* retinoic acid (ATRA) (72 h). Surface ICAM-1 expression was measured by flow cytometry. Data represent the mean fold change in ICAM-1 expression relative to the DMSO control (±SD). A one-way ANOVA with Dunnett's multiple comparisons test was performed. (B) Representative flow cytometry histograms of surface ICAM-1 expression on control- (DMSO; 72 h; black line) and ATRA- (1 μM; 72 h; red line) treated PDES compared to the isotype control (gray line). (C) Percentage cell viability of PDES (n = 5) following pre-treatment described in (B) before infection with CVA21 at increasing doses. Cell viability was measured by CellTiter-Glo 48 h post-infection. Data are presented as the mean percentage viability relative to the DMSO- or ATRA-alone condition (±SD). A two-way ANOVA with Šídák's multiple comparisons test was performed. (D) Percentage of live PDES (n = 5) following pre-treatment described in (B) before infection with CVA21 (MOI 0.5). PDES were harvested for LIVE/DEAD flow cytometry 48 h post-infection. Data represent the mean percentage of live cells within the total cell population (±SD). A two-way ANOVA with Fisher's multiple comparisons test was performed. (E) As described in (C) but assessing the percentage cell viability of PDES 3D spheroids (n = 4). (F) Percentage cell viability of PDES (n = 5) following pre-treatment with IFNα (5 ng/mL; 24 h), TNF (5 ng/mL; 24 h), or ATRA (1 μM or DMSO control; 72 h) alone or in combination before infection with CVA21 (MOI 0.1). Cell viability was measured by CellTiter-Glo post-infection. Data are presented as the mean percentage viability relative to the DMSO- or ATRA-alone control (±SD). A two-way ANOVA with Šídák's multiple comparisons test was performed to determine statistically significant differences from the CVA21-alone control. (G) Percentage cell viability of PDES (n = 6) following pre-treatment with ATRA (1 μM or vehicle control; 72 h) before treatment with PBMC-CM or CVA21-stimulated PBMC-CM for a further 24 h. PDES were then infected with CVA21 (MOI 0.1; 48 h). For PBMC-CM, n = 3 donors; CVA21 stimulation with MOI 0.1 for 48 h; 1:1 RPMI:CM. Cell viability was measured by CellTiter-Glo 48 h post-infection. Data are presented as the mean percentage viability of cells relative to DMSO- or ATRA-alone control (±SD). A two-way ANOVA with Šídák's multiple comparisons test was performed.

and presents CVA21 as a potential ICAM-1-dependent immunotherapy for patients with EWS, given its ability to infect and lyse ICAM-1-positive EWS cells and activate anti-tumor immune responses. Thus, CVA21 could provide an alternative treatment approach aimed at targeting a population of EWS cells associated with worse prognosis.

Inter- and intratumoral heterogeneity may arise due to varying expression/activity of the EWSR1::FLI1 fusion between cells or fluctuations over time within the same cell.[7] The expression of ICAM-1 in EWS has been previously inversely associated with EWSR1::FLI1 oncoprotein expression.[7] For example, EWSR1::FLI1^{low} cells have higher ICAM-1 and a more invasive phenotype, while EWSR1::FLI1^{high} cells express low ICAM-1[7] and present with an undifferentiated and rapidly proliferating phenotype. Critically, ICAM-1 has been identified as a repressed target of EWSR1::FLI1, hence the inverse relationship between ICAM-1 expression and EWSR1::FLI1 expression in EWS cells.[7,36] These data are corroborated by our ICAM-1-related findings, as the PDES used in this study have been previously characterized with heterogeneous EWSR1::FLI1 activity.[31] Importantly, while there was

clear heterogeneity in ICAM-1 expression within and between the PDES samples, the PDES isolated and used within this study all expressed ICAM-1. Given the relatively low abundance of ICAM-1-positive cells in tumors, but their enrichment within the PDES panel, it is possible that subpopulations of cells are enriched during the isolation process. For example, ICAM-1 may facilitate better adherence of EWSR1::FLI1^{low} EWS cells upon tissue dissociation and culture on tissue culture plastic, compared to EWSR1::FLI1^{high} with low/absent ICAM-1. Furthermore, the PDES panel generally displays a mesenchymal-like phenotype with increased expression of other adhesion molecules, including neurexin-1 (a synaptic adhesion protein) and extracellular matrix (ECM) proteins (fibrillin-1 and collagen), which could bias adhesion to tissue culture plastic and better enrichment of EWSR1::FLI1^{low} PDES.[28] However, it is important to note that upon detailed characterization, isolated PDES were consistent with their matched tumors for EWSR1 fusion breakpoints, EWSR1 fusion target gene expression, and mutational profile (including single-nucleotide polymorphisms within ICAM-1 exons). The decreased proliferation and Ki67 expression of PDES was also consistent with levels of Ki67 in paired tumors, reflecting their inherent resistance to doxorubicin, etoposide, vincristine, and ionizing radiation.[31] Finally, PDES did not respond to zoledronic acid in accordance with the clinical experience of patients.[50] Therefore, PDES represent a clinically relevant model to support pre-clinical evaluation of candidate EWS therapies, such as CVA21.

Exposure to pro-inflammatory cytokines can increase ICAM-1 expression on EWS cell lines via monocyte-secreted factors, including TNF,[39] and T cell-secreted IFNγ, independently of EWSR1::FLI1.[36] These findings align with our data (Figure S8A), whereby PDES exposed to cytokine-rich CVA21-stimulated PBMC-CM upregulated ICAM-1 expression. This suggests that ICAM-1 expression could be increased on EWS cells within the TME following CVA21 infection due to the induction of pro-inflammatory antiviral cytokines, thus increasing direct CVA21 oncolysis. Moreover, cytokine production could also promote immune cell recruitment, increasing immunogenicity and immune-mediated killing. As demonstrated herein, immune killing was largely NK cell mediated; however, uptake and presentation of EWS-specific antigens[9,10,51] in the presence of immunological danger (e.g., CVA21) could aid priming of EWS-specific T cells. Interestingly, CVA21-induced EWS-specific T cells could also sensitize EWS to clinically approved immune checkpoint inhibitors, as observed previously for other cancers.[52–55] Indeed, previous studies have demonstrated that EWSR1::FLI1^{low} cells are more resistant to T cell-mediated apoptosis, due to increased immune checkpoint expression.[36] Therefore, increased ICAM-1 expression[36] would enhance direct CVA21 oncolysis, while combination with immune checkpoint inhibitors could increase T cell-mediated killing. In support of this type of approach in the future, CVA21 has previously undergone phase 1/2 clinical testing in combination with pembrolizumab for advanced solid cancers.[27,56]

In contrast to PDES, EWS cell lines (i.e., EWSR1::FLI1^{high}) lack sufficient ICAM-1 and remain insensitive to CVA21 without pre-treatment with TNF or ATRA. Encouragingly, a subset of cells within EWS cell lines were sensitized to CVA21 oncolysis following ICAM-1 induction with TNF or ATRA pre-treatment. These findings suggest that patients with low ICAM-1 could be sensitized to CVA21 with ICAM-1-inducing combination treatments. Additionally, oncolytic viro-immunotherapy could be an effective strategy for treating EWS tumors despite the presence of populations of ICAM-1^{low} cells due to its immunostimulatory properties and ability to stimulate NK-mediated killing. We have previously demonstrated that oncolytic Maraba virus-stimulated PBMCs can kill EWS cell lines and PDES via NK cells.[41] Furthermore, the EWSR1::FLI1^{high} populations of EWS cells are typically more sensitive to chemotherapy and radiotherapy compared to the EWSR1::FLI1^{low} cells,[28] while EWSR1::FLI1^{low} cells have been reported to be more resistant to T cell-mediated killing,[36] radiotherapy, and chemotherapy.[29,31] Therefore, CVA21 treatment offers an attractive opportunity to eradicate these resistant cells (via direct oncolysis and/or NK cell-mediated killing), thus reducing disease relapse and metastasis.[34]

ATRA is a derivative of vitamin A that is well tolerated in patients and is frequently administered for skin conditions, such as acne, and to induce cellular differentiation in cancer treatment, including in acute promyelocytic leukemia.[46] Additionally, ATRA could have additional benefits to immunotherapy besides increasing ICAM-1 expression. For example, ATRA can deplete immunosuppressive myeloid-derived suppressor cells (MDSCs) in sarcoma xenograft models, leading to enhanced chimeric antigen receptor (CAR) T cell activity in both osteosarcoma and EWS.[36] Therefore, its widespread clinical use, availability, and safety indicate that ATRA would be suitable for combination therapy alongside CVA21 to induce ICAM-1 expression in EWS cells. Furthermore, in addition to combination with ATRA, CVA21 could complement existing radiotherapy treatment, as irradiated EWS cells can produce TNF following exposure to ionizing radiation.[57,58] Given our findings that TNF significantly enhances ICAM-1 expression and CVA21 oncolysis in PDES, local radiotherapy could stimulate intratumoral TNF secretion, increase ICAM-1 expression, and enhance CVA21 sensitivity. Interestingly, in other cancer settings, synergistic responses have been reported for OV in combination with radiotherapy due to increased oncolysis, inhibition of DNA repair, and an enhanced immune response.[59–61] Systemic delivery of ATRA may cause wider upregulation of ICAM-1 beyond the TME, while local radiotherapy would upregulate ICAM-1 within the TME and restrict CVA21 replication to the tumor and not wider tissues. However, this would limit the use of this combination strategy to localized EWS rather than widespread metastatic and/or relapsed disease. Further work should investigate CVA21 in combination with radiation and explore possible synergistic and/or inhibitory effects following combination with standard-of-care chemotherapy and second-line treatments, such as those being evaluated in patients with relapsed or refractory EWS.

We propose that CVA21, alone or in combination with ICAM-1-inducing agents, could provide a much-needed treatment option

for patients with EWS with metastatic disease, where increased ICAM-1 expression is associated with poor prognostic outcomes.[34] However, given the association between ICAM-1 and metastatic EWS, it is important to consider whether the ICAM-1-inducing agent could promote invasion and metastasis. Critically, the role of ICAM-1 in tumor progression is likely to be complex, with dual functional roles. For example, ICAM-1 can (1) promote immune cell interactions to better eradicate tumor cells[36] and (2) increase EWS invasion and metastasis.[34] Therefore, future work should determine whether increased ICAM-1 facilitates more effective immune-mediated killing and/or increased EWS migratory capacity. Early-phase clinical trials and appropriate *in vivo* models would help us address these remaining questions and determine whether the potential benefits of treatment supersede any negative effects. Interestingly, radiotherapy is used to treat EWS and can induce TNF,[57,58] which could ultimately enhance CVA21 susceptibility, something we would like to explore in the future. However, the induction of TNF (and/or other inflammatory cytokines) following radiotherapy has, to our knowledge, not been associated with increased metastatic disease. Therefore, potential treatments, such as those proposed herein, should be investigated in early-phase clinical trials, focusing on patients with metastatic and/or relapsed/refractory disease and a poor prognosis.

Importantly, our previous work in non-malignant cells suggests that additional cellular factors, in addition to ICAM-1 expression, are required for CVA21 replication/oncolysis, as increased ICAM-1 expression on naive B cells did not sensitize them to CVA21 direct oncolysis.[42] Other potential factors could include the expression of a decay-accelerating factor (DAF) that helps sequester CVA21 to the cell surface.[62] Interestingly, RNA sequencing data suggested that DAF expression is significantly higher in PDES (log2fold change of 3.5, adjusted $p = 2.98e{-}8$; data not shown) compared to EWS cell lines, which could facilitate CVA21 sequestration. In addition, it is also possible that TNF/ATRA treatment could affect downstream antiviral signaling and enhance CVA21 sensitivity. However, preliminary investigations exploring this possibility, through assessment of STAT1 phosphorylation, suggested that TNF/ATRA treatment did not abrogate the antiviral IFN response to promote CVA21 susceptibility (Figure S8B). By contrast, the antiviral STAT1 phosphorylation response was significantly enhanced upon TNF treatment, opposing the observed enhancement of CVA21 oncolysis in the presence of TNF. Naturally, we cannot exclude additional modulation of downstream antiviral IFN signaling; however, these initial observations, and the correlation analysis, suggest that ICAM-1 may be the predominant factor responsible for CVA21 sensitivity in EWS.

Our data demonstrate that PDES do not produce type I IFN in response to CVA21 infection *in vitro*, while PBMCs produce an array of antiviral cytokines. The production of pro-inflammatory cytokines, such as TNF and IFNα, is essential in the generation of robust antiviral and anti-tumor immune responses.[19,42] Hence, the expression of cytokines in response to CVA21 by PBMCs may not abrogate overall anti-tumor efficacy, despite the inhibitory effects on viral oncolysis observed herein. Therefore, rather than inhibiting the immune action of these cytokines, navigating around the antiviral response by pharmacologically upregulating ICAM-1 expression could maintain CVA21 oncolytic potency and the induction of anti-tumor immune responses.

While CVA21 has not yet been trialed in EWS, a handful of patients with EWS have been enrolled in basket pediatric cancer OV trials, which have all demonstrated acceptable safety levels. Moreover, one patient with EWS showed potential evidence of efficacy after intratumoral injection of oncolytic vaccinia virus[22]; one patient with metastatic EWS showed stable disease following intravenous administration of HSV1716,[20] while another patient with metastatic EWS was given HSV1716 via intratumoral administration directly to a lung metastatic site.[21] These examples are limited for EWS; however, they demonstrate that OVs can be delivered either systemically or directly to the tumor, including at some metastatic sites, providing a range of delivery options for CVA21. Importantly, CVA21 has been safe and well tolerated in patients and has been delivered by multiple routes, including intravenously,[25,27,63] intravesicularly,[55] or intratumorally,[64,65] indicating that CVA21 could be administered to patients with EWS in a variety of ways. Critically, pre-existing CVA21 immunity did not appear to limit its efficacy,[65] further supporting its therapeutic potential for patients with EWS with or without prior CVA21 exposure.

This study provides evidence of CVA21 being an attractive candidate therapy targeting ICAM-1-expressing EWS tumor cells, a population of cells associated with worse prognoses and metastasis.[34] The ability of CVA21 to eradicate ICAM-1-expressing EWS cells by direct oncolysis and immune activation makes CVA21 an attractive candidate for the treatment of EWS, thus providing an alternative approach to treat relapsed or metastatic disease.

MATERIALS AND METHODS

Cell culture and reagents

The procedure of PDES isolation and characterization has been previously described.[28,31] Informed consent and ethical approval for the collection of the tumors and generation of PDES were obtained through GenoEWING (IRAS 167880, EDGE 79301). All EWS cell lines contain EWSR1 gene rearrangements and CD99 surface expression. EWS cell lines and PDES were routinely tested for mycoplasma using the EZ-PCR mycoplasma test kit (Geneflow, Lichfield, UK). EWS cell lines were cultured in Roswell Park Memorial Institute (RPMI)-1640 medium (Gibco, Thermo Fisher Scientific, Waltham, MA, USA) supplemented with 10% fetal bovine serum (FBS; Sigma-Aldrich, St. Louis, MO, USA); PDES were cultured in the same medium also supplemented with 1% penicillin/streptomycin (Gibco, Thermo Fisher Scientific). Mel624 cells were cultured in Dulbecco's modified Eagle's medium (DMEM), supplemented with 10% FBS. 2′-C-methylcytidine (Thermo Fisher Scientific) was dissolved in Hank's balanced salt solution (HBSS; Thermo Fisher Scientific) and used at a final concentration of 100 μM. ATRA (Sigma-Aldrich) was dissolved to a concentration of 10 mM in dimethyl

sulfoxide (DMSO; Sigma-Aldrich). For pre-treatments, PDES were pre-treated with 5 ng/mL recombinant human IFN-α2 (R&D Systems, MN, USA) or recombinant human TNF (R&D Systems) for 24 h or 1 μM ATRA (Sigma-Aldrich) for 72 h. Cytokines or ATRA were removed prior to CVA21 treatment in fresh medium.

PDES and EWS cell lines used throughout this study are identified by unique symbols in each graph (Figure 1E). PDES derived from diagnostic biopsy tissue (n = 5) are represented as follows: CCRG1-L-008, diamond; CCRG1-L-014, inverted triangle; CCRG1-L-017, circle; CCRG1-L-023, square; and CCRG1-L-066, triangle. PDES derived from relapsed tissue samples (n = 2) are represented as follows: CCRG1-L-087, hollow square, and CCRG1-L-088, hollow circle. EWS cell lines are identified by unique symbols: TC-32, square with cross; SK-N-MC, diamond with cross; TC-71, circle with cross; ES-5838, cross; SK-ES-1, 8-pointed cross; A673, plus; and TTC-446, hexagon with cross.

Processing of human PBMCs

PBMCs were obtained from leukocyte apheresis cones supplied by the National Health Service (NHS) Blood and Transplant service. PBMCs were isolated using Lympholyte (VH Bio, Gateshead, UK) and density centrifugation.

ICAM-1 Kaplan-Meier analyses

Kaplan-Meier (KM) survival analyses were performed as previously described.[28] Briefly, results were linked to clinical outcome data in R (v.4.5.1), and the prognostic value of *ICAM1* was examined in publicly available RNA datasets of diagnostic ES tissues (GEO: GSE63157, GSE17618, and GSE17674), using the Cox proportional hazards regression model, and the optimal cut point in the data was determined using Harrell's C index.[66] The Cox model was then performed using the defined cut point, and KM plots were generated using Survminer and ggplot.

Flow cytometry analysis

A Cytoflex LX (Beckman Coulter, Brea, CA, USA) was used for data acquisition and analyzed using CytExpert v.2.6. Flow cytometry-based experiments are described in more detail within the relevant section below.

ICAM-1 expression

Total RNA sequencing was performed as previously described.[28] For ICAM-1 IHC, fresh frozen EWS tissue sections (5 μm) were fixed in fresh 4% PFA for 15 min before three washes with PBS. Access to EWS and normal tissue was covered under the following ethics: (1) IRAS 167880 and EDGE 79301 for tumors with paired PDES and (2) VIVO Biobank (23/EM/0130 IRAS 327843) for all other tumors and normal tissue. Tissue was stained with the ICAM-1 IH-C*easy* ICAM-1 Ready-To-Use IHC Kit (Proteintech, Manchester, UK) according to the manufacturer's instructions. Mouse IgG2b (MPC-11; Proteintech) was used as an isotype control (0.2 μg/mL). Images were captured using a Zeiss Axio Imager microscope fitted with an ApoTome module and an Axiocam camera (Zeiss, Germany). A fresh-frozen healthy human lymph node was used as a positive control, and cytospins of TC-32 EWS cells were used as an ICAM-1-low/negative control. Relative ICAM-1 staining was measured using ImageJ (v.1.54p). ICAM-1 staining was quantified using ImageJ analysis; each image was deconvoluted separately into RGB for the DAB signal (brown) and hematoxylin nuclear counterstain (blue) using brightness/contrast thresholds set based on positive and negative control samples. Each image was then converted to a binary mask, and the mean intensity for each signal was quantified separately on each image. The ICAM-1 staining score was calculated as the ratio of DAB signal to nuclear staining signal for each image. $n \geq 3$ images were taken for each section on 40× magnification. ICAM-1 scores were then normalized by subtracting the mean staining score from tissue-matched isotype-stained samples.

For ICAM-1 surface protein expression, cells were harvested with trypsin:ethylenediaminetetraacetic acid (EDTA) solution (Sigma-Aldrich) and stained with mouse anti-human ICAM-1 (CD54)-PE antibody at 20 μL per 10^6 cells (3 μg/mL; clone LB-2, #347977, BD Bioscience, UK) or an equivalent concentration of mouse IgG2b-PE isotype control (clone 27-35, #555743, BD Bioscience) diluted in fluorescence-activated cell sorting (FACS) buffer (PBS supplemented with 2 mM EDTA and 2% FBS) for 30 min at 4°C, washed with PBS, and fixed with 1% PFA. Expression was quantified by flow cytometry, and relative expression was determined as the fold change in mean fluorescence intensity (MFI) of ICAM-1 versus the isotype control.

CVA21 replication and concentration

Wild-type CVA21 (*Kyukendall* strain) was propagated in-house from stocks purchased from ATCC (ATCC VR-850; ATCC, Manassas, WA, USA). The virus was propagated by harvesting Mel624 cell supernatants following infection with CVA21 at 0.001 plaque-forming units/cell for 72 h. CVA21 particles were initially pelleted from the supernatant by centrifugation at 36,000 rpm for 2 h (SW45 rotor, Optima L-80 ultracentrifuge, Beckman Coulter) before being purified by OptiPrep density gradient centrifugation (35%–15% gradient) at 36,000 rpm for 1.5 h (SW41 Ti rotor). Bands containing CVA21 were collected, aliquoted, and stored at −80°C. To assess CVA21 replication in EWS cells, cells were infected with CVA21 at the stated MOI for 16 h, after which cells and supernatant were collected and immediately frozen at −80°C. CVA21-containing samples underwent 2 freeze/thaw cycles. Titer of viral stock was determined by plaque assay.

CVA21 plaque assays

Mel624 cells were seeded in 6-well plates overnight before infection with 1 mL CVA21-containing samples serially diluted 10-fold in serum-free DMEM. For EWS CVA21 replication experiments, Mel624 cells were also infected with CVA21 stock at the originally stated MOI and defined as the "input." After 2 h of infection, virus-containing medium was replaced with overlay medium (1:2 ratio of 10% DMEM with 1.6% carboxymethylcellulose). Cells were cultured for 48–72 h before overlay medium was removed,

and cells were washed and fixed in 1% PFA solution. Cells were counterstained using 1% (w/v) methylene blue, and viral-induced plaques were counted manually. Viral titers were calculated as the number of plaques multiplied by the dilution factor. Relative viral titers were measured as fold change in viral titer from experimental samples compared to input samples.

CellTiter-Glo viability assays

Cell viability assays were performed by seeding EWS cell lines or PDES at 5e3 cells per well in a 96-well plate for 2D monolayers and a 96-well ultra-low adherence plate (Corning, NY, USA) for 3D spheroids. Cell viability was determined using the CellTiter-Glo luminescent cell viability assay (Promega, Madison, WI, USA) according to the manufacturer's instructions, and luminescence was measured using an Agilent BioTek Cytation 5 plate reader (Agilent Technologies, Santa Clara, CA, USA) with a 1 s integration time. Relative viability of treated samples was measured as the mean luminescence signal from $n \geq 3$ technical repeats relative to untreated or DMSO-treated controls. For ATRA experiments, treated samples were normalized relative to ATRA alone to demonstrate the effect of CVA21, thus normalizing for the cytostatic effects of ATRA.

High-content imaging

PDES culture medium containing floating dead cells was removed, and cells were washed with PBS before being fixed with 4% PFA for 15 min, permeabilized with 0.1% Triton X, and stained with 4′,6-diamidino-2-phenylindole (DAPI; 0.2 μg/mL, Sigma-Aldrich) and TOTO-3 Iodide (500 nM; Thermo Fisher Scientific) for 30 min. Images were captured using the Cell Discoverer 7 High-Content Imaging fluorescent microscope (CD7; Carl Zeiss, Cambridge, UK). $n = 3$ fields of view were captured for each well at 10× magnification. Cell number was assessed by counting the number of DAPI-stained nuclei in each field of view using Zen Blue Imaging Software (v.3.6, Zeiss).

NK cell depletion

For NK cell depletion experiments, NK cells were isolated using anti-human $CD56^+$ MicroBeads (#130-050-401, Miltenyi Biotech, Bergisch Gladbach, Germany) and LS columns (#130-042-401, Miltenyi Biotech) according to the manufacturer's instructions. NK cell depletion was confirmed for each PBMC donor by staining whole PBMCs, NK-depleted PBMCs, and enriched NK cells with anti-human CD45-APC (Miltenyi Biotech), CD56-eF450 (Thermo Fisher Scientific), CD3-PerCP (BioLegend, San Diego, CA, USA), and LIVE/DEAD Fixable Dead Cell Stain Kit (Invitrogen, Thermo Fisher Scientific) diluted in FACS buffer for 30 min at 4°C; cells were washed with PBS and fixed with 1% PFA prior to flow cytometry analysis.

PBMC-mediated killing assays

For short-term PBMC killing assays, PBMCs were stimulated with CVA21 (MOI 0.1), or left untreated, for 48 h. After 48 h, freshly isolated PBMCs were co-cultured with PDES stained with CellTracker Green CMFDA Dye (Invitrogen, Thermo Fisher Scientific) in a 96-well round-bottom plate for 5 h; cells were cultured in suspension at a 25:1 PBMC:PDES ratio. When NK cell-depleted PBMCs were used, NK cells were depleted as described above immediately prior to co-culture with target EWS cells. Co-cultures were then stained with LIVE/DEAD Fixable Dead Cell Stain Kit (Invitrogen, Thermo Fisher Scientific) for 30 min at 4°C, washed with PBS, and fixed with 1% PFA prior to flow cytometry acquisition and analysis.

For adherent PBMC killing assays, PDES or EWS cell lines were seeded and left to adhere overnight in 24-well plates. The following day, EWS cells were infected with CVA21 (MOI 0.5), or left untreated, for 2 h before co-culture with freshly isolated PBMCs at a 10:1 PBMC:EWS cell ratio for 48 h. When NK cell-depleted PBMCs were used, NK cells were depleted as described above immediately prior to co-culture with target EWS cells. Total cell co-cultures, including detached dead cells, were then harvested using trypsin:EDTA solution and stained with the LIVE/DEAD Fixable Dead Cell Stain Kit and CD45-APC (Miltenyi Biotech) for 30 min at 4°C; cells were washed with PBS and fixed with 1% PFA prior to flow cytometry analysis.

PBMC-CMs

To generate PBMC-CMs, PBMCs were either left untreated or stimulated with CVA21 (MOI 0.1) for 48 h before cells were harvested and pelleted, medium collected, and virus removed by filtration through an Amicon Ultra Filter 100 kDa molecular weight cutoff filter (Merck Millipore, Burlington, MA, USA). PBMC-CM was stored at −20°C. For PBMC-CM pre-treatment, PDES were treated with PBMC-CM mixed 1:1 with RPMI-1640 plus 10% FBS for 24 h before further treatments.

Phospho-STAT-1 staining

Cells were pre-treated with TNF (5 ng/mL; 24 h) or ATRA (1 μM; or DMSO control; 72 h) or left untreated before stimulating with IFNα (5 ng/mL), PBMC-CM, or CVA21-stimulated PBMC-CM (1:1 RPMI:CM) for 30 min. Cells were then immediately dissociated, transferred to FACS tubes, and fixed with 1% PFA for 15 min at 37°C. Cells were then washed twice with ice-cold FACS buffer, permeabilized with pre-chilled True-Phos Perm Buffer (#425401 BioLegend) for 1 h at −20°C, washed two more times with FACS buffer, and stained with mouse anti-human STAT1 Phospho Y701 PE antibody at 5 μL per e6 cells (2.5 μg/mL; clone A17012A, #666403, BioLegend) or an equivalent concentration of mouse IgG1 PE isotype (clone MOPC-21, #400140) diluted in FACS buffer for 30 min at 4°C. Cells were washed twice more with FACS buffer before immediate acquisition by flow cytometry. Relative expression was determined as fold change in MFI for phospho-STAT-1 versus the isotype control.

Statistical analysis

Differential expression of RNAs between cell lines and PDES was identified using DESeq2.[35] Other statistical analyses were performed using GraphPad PRISM 10.5.0 (GraphPad Software, San Diego, CA, USA). Paired analyses were used when assessing treatments for each PDES, EWS cell line, and individual PBMC donor. For each

experiment, figure legends provide the details of (1) the individual statistical test (e.g., *t* test or one-way or two-way analysis of variance [ANOVA]) used, (2) the post hoc multiple comparisons test used, and (3) the number of biological repeats performed. Error bars represent mean ± standard deviation.

DATA AND CODE AVAILABILITY

The FASTQ files of the RNA sequencing data of PDES are available in the Research Data Leeds Repository (University of Leeds).[67,68]

ACKNOWLEDGMENTS

The authors extend thanks to the patients, parents, and legal guardians who consented and donated samples for this research. Additional thanks are extended to the Leeds Institute of Medical Research (LIMR) flow cytometry and imaging services for their assistance with the project. The authors would also like to thank the following charities for their funding and support. Firstly, we would like to thank Sarcoma UK (SUK10.2021 and SUK8.2024) for funding R.T.B. Secondly, we would like to thank the Bone Cancer Research Trust (BCRT) for funding both T.B. (BCRT/6118 and BCRT/11725) and E.A.R. (BCRT 5918 and BCRT/36/13). Finally, we would like to thank Child Health Outcomes Research at Leeds (CHORAL) for funding F.I. All other authors were supported by their respective affiliation/institution. The views expressed are those of the authors and not necessarily those of the NHS, CHORAL, or the funders of this work. The graphical abstract was created with BioRender.com.

AUTHOR CONTRIBUTIONS

Data curation, R.T.B., T.B., E.A.R., and M.V.; formal analysis, R.T.B., T.B., E.A.R., and M.V.; funding acquisition, R.T.B., T.B., E.A.R., S.A.B., and F.E.-M.; investigation, R.T.B. and T.B.; methodology, R.T.B., T.B., G.P.C., and F.E.-M.; project administration, R.T.B.; resources, R.T.B., E.A.R., F.I., S.A.B., and F.E.-M.; validation, R.T.B.; visualization, R.T.B.; conceptualization, R.T.B., T.B., and F.E.-M.; writing – original draft, R.T.B., G.P.C., and F.E.-M.; writing – review & editing, R.T.B., T.B., E.A.R., G.P.C., F.I., M.V., S.A.B., and F.E.-M.; supervision, G.P.C., S.A.B., and F.E.-M.

DECLARATION OF INTERESTS

The authors declare no competing interests.

SUPPLEMENTAL INFORMATION

Supplemental information can be found online at https://doi.org/10.1016/j.omton.2026.201367.

REFERENCES

1. Zöllner, S.K., Amatruda, J.F., Bauer, S., Collaud, S., de Álava, E., DuBois, S.G., Hardes, J., Hartmann, W., Kovar, H., Metzler, M., et al. (2021). Ewing Sarcoma—Diagnosis, Treatment, Clinical Challenges and Future Perspectives. J. Clin. Med. *10*, 1685. https://doi.org/10.3390/jcm10081685.

2. Digklia, A., Dolcan, A., Kucharczyk, M.A., Jones, R.L., and Napolitano, A. (2023). Optimal Delivery of Follow-Up Care Following Treatment for Adults Treated for Ewing Sarcoma. Cancer Manag. Res. *15*, 537–545. https://doi.org/10.2147/CMAR.S362693.

3. Apfelbaum, A.A., Wrenn, E.D., and Lawlor, E.R. (2022). The importance of fusion protein activity in Ewing sarcoma and the cell intrinsic and extrinsic factors that regulate it: A review. Front. Oncol. *12*, 1044707. https://doi.org/10.3389/fonc.2022.1044707.

4. Delattre, O., Zucman, J., Plougastel, B., Desmaze, C., Melot, T., Peter, M., Kovar, H., Joubert, I., de Jong, P., Rouleau, G., et al. (1992). Gene fusion with an ETS DNA-binding domain caused by chromosome translocation in human tumours. Nature *359*, 162–165. https://doi.org/10.1038/359162a0.

5. Aynaud, M.M., Mirabeau, O., Gruel, N., Grossetête, S., Boeva, V., Durand, S., Surdez, D., Saulnier, O., Zaïdi, S., Gribkova, S., et al. (2020). Transcriptional Programs Define Intratumoral Heterogeneity of Ewing Sarcoma at Single-Cell Resolution. Cell Rep. *30*, 1767–1779.e6. https://doi.org/10.1016/j.celrep.2020.01.049.

6. Goodspeed, A., Bodlak, A., Duffy, A.B., Nelson-Taylor, S., Oike, N., Porfilio, T., Shirai, R., Walker, D., Treece, A., Black, J., et al. (2025). Single-Cell RNA Sequencing of Ewing Sarcoma Tumors Demonstrates Transcriptional Heterogeneity and Clonal Evolution. Clin. Cancer Res. *31*, 2010–2023. https://doi.org/10.1158/1078-0432.CCR-24-2040.

7. Franzetti, G.A., Laud-Duval, K., Van Der Ent, W., Brisac, A., Irondelle, M., Aubert, S., Dirksen, U., Bouvier, C., De Pinieux, G., Snaar-Jagalska, E., et al. (2017). Cell-to-cell heterogeneity of EWSR1-FLI1 activity determines proliferation/migration choices in Ewing sarcoma cells. Oncogene *36*, 3505–3514. https://doi.org/10.1038/onc.2016.498.

8. Crompton, B.D., Stewart, C., Taylor-Weiner, A., Alexe, G., Kurek, K.C., Calicchio, M.L., Kiezun, A., Carter, S.L., Shukla, S.A., Mehta, S.S., et al. (2014). The genomic landscape of pediatric Ewing sarcoma. Cancer Discov. *4*, 1326–1341. https://doi.org/10.1158/2159-8290.CD-13-1037.

9. Morales, E., Olson, M., Iglesias, F., Dahiya, S., Luetkens, T., and Atanackovic, D. (2020). Role of immunotherapy in Ewing sarcoma. J. Immunother. Cancer *8*, e000653. https://doi.org/10.1136/jitc-2020-000653.

10. Altvater, B., Kailayangiri, S., Theimann, N., Ahlmann, M., Farwick, N., Chen, C., Pscherer, S., Neumann, I., Mrachatz, G., Hansmeier, A., et al. (2014). Common Ewing sarcoma-associated antigens fail to induce natural T cell responses in both patients and healthy individuals. Cancer Immunol. Immunother. *63*, 1047–1060. https://doi.org/10.1007/s00262-014-1574-3.

11. Kumar, R.R., Agarwal, N., Shree, A., Gorain, J.K., Rahul, E., Ganguly, S., Bakhshi, S., and Sharma, U. (2025). Decoding the immune landscape in Ewing sarcoma pathogenesis: The role of tumor infiltrating immune cells and immune milieu. J. Bone Oncol. *52*, 100678. https://doi.org/10.1016/j.jbo.2025.100678.

12. Stahl, D., Gentles, A.J., Thiele, R., and Gütgemann, I. (2019). Prognostic profiling of the immune cell microenvironment in Ewing's Sarcoma Family of Tumors. Oncoimmunology *8*, e1674113. https://doi.org/10.1080/2162402X.2019.1674113.

13. Evdokimova, V., Gassmann, H., Radvanyi, L., and Burdach, S.E.G. (2022). Current State of Immunotherapy and Mechanisms of Immune Evasion in Ewing Sarcoma and Osteosarcoma. Cancers (Basel) *15*, 272. https://doi.org/10.3390/cancers15010272.

14. Tawbi, H.A., Burgess, M., Bolejack, V., Van Tine, B.A., Schuetze, S.M., Hu, J., D'Angelo, S., Attia, S., Riedel, R.F., Priebat, D.A., et al. (2017). Pembrolizumab in advanced soft-tissue sarcoma and bone sarcoma (SARC028): a multicentre, two-cohort, single-arm, open-label, phase 2 trial. Lancet Oncol. *18*, 1493–1501. https://doi.org/10.1016/S1470-2045(17)30624-1.

15. Berghuis, D., de Hooge, A.S., Santos, S.J., Horst, D., Wiertz, E.J., Van Eggermond, M.C., Van Den Elsen, P.J., Taminiau, A.H., Ottaviano, L., Schaefer, K.L., et al. (2009). Reduced human leukocyte antigen expression in advanced-stage Ewing sarcoma: Implications for immune recognition. J. Pathol. *218*, 222–231. https://doi.org/10.1002/path.2537.

16. Spurny, C., Kailayangiri, S., Jamitzky, S., Altvater, B., Wardelmann, E., Dirksen, U., Hardes, J., Hartmann, W., and Rossig, C. (2018). Programmed cell death ligand 1 (PD-L1) expression is not a predominant feature in Ewing sarcomas. Pediatr. Blood Cancer *65*. https://doi.org/10.1002/pbc.26719.

17. Machado, I., López-Guerrero, J.A., Scotlandi, K., Picci, P., and Llombart-Bosch, A. (2018). Immunohistochemical analysis and prognostic significance of PD-L1, PD-1, and CD8+ tumor-infiltrating lymphocytes in Ewing's sarcoma family of tumors (ESFT). Virchows Arch. *472*, 815–824. https://doi.org/10.1007/s00428-018-2316-2.

18. Chaurasiya, S., Chen, N.G., and Fong, Y. (2018). Oncolytic viruses and immunity. Curr. Opin. Immunol. *51*, 83–90. https://doi.org/10.1016/j.coi.2018.03.008.

19. Müller, L.M.E., Holmes, M., Michael, J.L., Scott, G.B., West, E.J., Scott, K.J., Parrish, C., Hall, K., Stäble, S., Jennings, V.A., et al. (2019). Plasmacytoid dendritic cells orchestrate innate and adaptive anti-tumor immunity induced by oncolytic coxsackievirus A21. J. Immunother. Cancer *7*, 164. https://doi.org/10.1186/s40425-019-0632-y.

20. Streby, K.A., Currier, M.A., Triplet, M., Ott, K., Dishman, D.J., Vaughan, M.R., Ranalli, M.A., Setty, B., Skeens, M.A., Whiteside, S., et al. (2019). First-in-Human Intravenous Seprehvir in Young Cancer Patients: A Phase 1 Clinical Trial. Mol. Ther. *27*, 1930–1938. https://doi.org/10.1016/j.ymthe.2019.08.020.

21. Streby, K.A., Geller, J.I., Currier, M.A., Warren, P.S., Racadio, J.M., Towbin, A.J., Vaughan, M.R., Triplet, M., Ott-Napier, K., Dishman, D.J., et al. (2017). Intratumoral injection of HSV1716, an oncolytic herpes virus, is safe and shows evidence of immune response and viral replication in young cancer patients. Clin. Cancer Res. *23*, 3566–3574. https://doi.org/10.1158/1078-0432.CCR-16-2900.

22. Cripe, T.P., Ngo, M.C., Geller, J.I., Louis, C.U., Currier, M.A., Racadio, J.M., Towbin, A.J., Rooney, C.M., Pelusio, A., Moon, A., et al. (2015). Phase 1 study of intratumoral Pexa-Vec (JX-594), an oncolytic and immunotherapeutic vaccinia virus, in pediatric cancer patients. Mol. Ther. *23*, 602–608. https://doi.org/10.1038/mt.2014.243.

23. Bradley, S., Jakes, A., Harrington, K., Pandha, H., Melcher, A., and Errington-Mais, F. (2014). Applications of coxsackievirus A21 in oncology. Oncolytic Virother. *47*, 47. https://doi.org/10.2147/ov.s56322.

24. Spickard, A., Evans, H., Knight, V., and Johnson, K. (1963). ACUTE RESPIRATORY DISEASE IN NORMAL VOLUNTEERS ASSOCIATED WITH COXSACKIE A-21 VIRAL INFECTION. III. RESPONSE TO NASOPHARYNGEAL AND ENTERIC INOCULATION. J. Clin. Investig. *42*, 840.

25. Pandha, H., Harrington, K., Ralph, C., Melcher, A., Grose, M., and Shafren, D. (2015). Phase I/II storm study: Intravenous delivery of a novel oncolytic immunotherapy agent, Coxsackievirus A21, in advanced cancer patients. J. Immunother. Cancer *3*, P341. https://doi.org/10.1186/2051-1426-3-s2-p341.

26. Andtbacka, R.H.I., Curti, B.D., Kaufman, H., Daniels, G.A., Nemunaitis, J.J., Spitler, L.E., Hallmeyer, S., Lutzky, J., Schultz, S.M., Whitman, E.D., et al. (2015). Final data from CALM: A phase II study of Coxsackievirus A21 (CVA21) oncolytic virus immunotherapy in patients with advanced melanoma. J. Clin. Oncol. *33*, 9030. https://doi.org/10.1200/jco.2015.33.15_suppl.9030.

27. Rudin, C.M., Pandha, H.S., Gupta, S., Zibelman, M.R., Akerley, W., Day, D., Hill, A.G., Sanborn, R.E., O'Day, S.J., Clay, T.D., et al. (2018). Phase Ib KEYNOTE-200: A study of an intravenously delivered oncolytic virus, coxsackievirus A21 in combination with pembrolizumab in advanced NSCLC and bladder cancer patients. Ann. Oncol. *29*, viii732. https://doi.org/10.1093/annonc/mdy424.050.

28. Roundhill, E.A., Chicon-Bosch, M., Jeys, L., Parry, M., Rankin, K.S., Droop, A., and Burchill, S.A. (2021). RNA sequencing and functional studies of patient-derived cells reveal that neurexin-1 and regulators of this pathway are associated with poor outcomes in Ewing sarcoma. Cell. Oncol. *44*, 1065–1085. https://doi.org/10.1007/s13402-021-00619-8.

29. Roundhill, E.A., Vasconcelos, E.J., Westhead, D.R., Grissenberger, S., Distel, M., and Burchill, S.A. (2023). Abstract 4683: Developing human Ewing sarcoma *in vitro* models to prioritise new treatments. Cancer Res. *83*, 4683. https://doi.org/10.1158/1538-7445.AM2023-4683.

30. Brookes, M.J., Roundhill, E.A., Jeys, L., Parry, M., Burchill, S.A., and Rankin, K.S. (2022). Membrane-type 1 matrix metalloproteinase as predictor of survival and candidate therapeutic target in Ewing sarcoma. Pediatr. Blood Cancer *69*, e29959. https://doi.org/10.1002/pbc.29959.

31. Roundhill, E.A., Vasconcelos, E.J.R., Davies, J., and Burchill, S.A. (2026). Characterisation of Bespoke Patient-Derived In Vitro Models of Ewing Sarcoma. Cancers (Basel) *18*, 512. https://doi.org/10.3390/cancers18030512.

32. Minas, T.Z., Surdez, D., Javaheri, T., Tanaka, M., Howarth, M., Kang, H.-J., Han, J., Han, Z.-Y., Sax, B., Kream, B.E., et al. (2017). Combined experience of six independent laboratories attempting to create an Ewing sarcoma mouse model. Oncotarget *8*, 34141–34163. https://doi.org/10.18632/oncotarget.9388.

33. Nanni, P., Landuzzi, L., Manara, M.C., Righi, A., Nicoletti, G., Cristalli, C., Pasello, M., Parra, A., Carrabotta, M., Ferracin, M., et al. (2019). Bone sarcoma patient-derived xenografts are faithful and stable preclinical models for molecular and therapeutic investigations. Sci. Rep. *9*, 12174. https://doi.org/10.1038/s41598-019-48634-y.

34. Pan, B., Bu, X., Cao, M., Zhang, X., Huo, T., Li, Z., Gao, X., Jing, L., Luo, X., Feng, H., et al. (2021). Inactivation of ICAM1 inhibits metastasis and improves the prognosis of Ewing's sarcoma. J. Cancer Res. Clin. Oncol. *147*, 393–401. https://doi.org/10.1007/s00432-020-03431-3.

35. Love, M.I., Huber, W., and Anders, S. (2014). Moderated estimation of fold change and dispersion for RNA-seq data with DESeq2. Genome Biol. *15*, 550. https://doi.org/10.1186/s13059-014-0550-8.

36. Bailey, K.M., Julian, C.M., Klinghoffer, A.N., Bernard, H., Lucas, P.C., and McAllister-Lucas, L.M. (2019). EWS-FLI1 low Ewing sarcoma cells demonstrate decreased susceptibility to T-cell-mediated tumor cell apoptosis. Oncotarget *10*, 3385–3399. https://doi.org/10.18632/oncotarget.26939.

37. Jamitzky, S., Altvater, B., Krekeler, C., Hoen, L., Brandes, C., Ebbinghaus, J., Richter, L., Kosel, L., Ochs, L., Farwick, N., et al. (2024). Ganglioside SSEA-4 in Ewing sarcoma marks a tumor cell population with aggressive features and is a potential cell-surface immune target. Sci. Rep. *14*, 11935. https://doi.org/10.1038/s41598-024-62849-8.

38. Gassmann, H., Thiede, M., Weiß, J., Biele, E., Flohé, L., Lachermaier, H., Prexler, C., Evdokimova, V., Radvanyi, L., Akhtar, I., et al. (2024). Cytokine screening identifies TNF to potentially enhance immunogenicity of pediatric sarcomas. Front. Immunol. *15*, 1347404. https://doi.org/10.3389/fimmu.2024.1347404.

39. Biele, E., Schober, S.J., Prexler, C., Thiede, M., Heyking, K.v., Gassmann, H., Eck, J., Xue, B., Burdach, S., and Thiel, U. (2021). Monocyte Maturation Mediators Upregulate CD83, ICAM-1 and MHC Class 1 Expression on Ewing's Sarcoma, Enhancing T Cell Cytotoxicity. Cells *10*, 3070. https://doi.org/10.3390/cells10113070.

40. Visser, L.L., Bleijs, M., Margaritis, T., van de Wetering, M., Holstege, F.C.P., and Clevers, H. (2023). Ewing Sarcoma Single-cell Transcriptome Analysis Reveals Functionally Impaired Antigen-presenting Cells. Cancer Res. Commun. *3*, 2158–2169. https://doi.org/10.1158/2767-9764.CRC-23-0027.

41. Barr, T., Jennings, V.A., Roundhill, E.A., Baugh, R.T., Yamrali, M., Owston, H.E., McGonagle, D., Giannoudis, P.V., Caplen, N.J., Khan, J., et al. (2025). Oncolytic Maraba Virus MG1 Mediates Direct and Natural Killer Cell-Dependent Lysis of Ewing Sarcoma. Cancers (Basel) *17*, 3319. https://doi.org/10.3390/cancers17203319.

42. Holmes, M., Scott, G.B., Heaton, S., Barr, T., Askar, B., Müller, L.M.E., Jennings, V.A., Ralph, C., Burton, C., Melcher, A., et al. (2023). Efficacy of coxsackievirus A21 against drug-resistant neoplastic B cells. Mol. Ther. Oncolytics *29*, 17–29. https://doi.org/10.1016/j.omto.2023.03.002.

43. Askar, B., Heaton, S., Barr, T., Baugh, R., Alzamel, N., McConnell, S., Jennings, V.A., Volpato, M., Ralph, C., Jain, M., et al. (2026). Apoptotic modulators enhance oncolytic virus-induced cytokine killing in acute myeloid leukaemia (AML). Br. J. Cancer *135*, 152–162. https://doi.org/10.1038/s41416-026-03417-x.

44. Tolomeo, M., Cavalli, A., and Cascio, A. (2022). STAT1 and Its Crucial Role in the Control of Viral Infections. Int. J. Mol. Sci. *23*, 4095. https://doi.org/10.3390/ijms23084095.

45. Balado-Simó, P., Morgado-Carrasco, D., Gómez-Armayones, S., López-Ferrer, A., Barco, D., Ferrándiz-Pulido, C., and Podlipnik, S. (2025). An Updated Review of Topical Tretinoin in Dermatology: From Acne and Photoaging to Skin Cancer. J. Clin. Med. *14*, 7958. https://doi.org/10.3390/jcm14227958.

46. Ni, X., Hu, G., and Cai, X. (2019). The success and the challenge of all-trans retinoic acid in the treatment of cancer. Crit. Rev. Food Sci. Nutr. *59*, S71–S80. https://doi.org/10.1080/10408398.2018.1509201.

47. Chen, C., Diao, D., Guo, L., Shi, M., Gao, J., Hu, M., Yu, M., Qian, L., and Guo, N. (2012). All-Trans-Retinoic Acid Modulates ICAM-1 N-Glycan Composition by Influencing GnT-III Levels and Inhibits Cell Adhesion and Trans-Endothelial Migration. PLoS One *7*, e52975. https://doi.org/10.1371/journal.pone.0052975.

48. Marchetti, M., Falanga, A., Giovanelli, S., Oldani, E., and Barbui, T. (1996). All-trans-retinoic acid increases adhesion to endothelium of the human promyelocytic leukaemia cell line NB4. Br. J. Haematol. *93*, 360–366. https://doi.org/10.1046/j.1365-2141.1996.4911029.x.

49. Liu, Y.I., Lee, M.-O., Wang, H.G., Li, Y., Hashimoto, Y., Klaus, M., Reed, J.C., and Zhang, X.-K. (1996). Retinoic Acid Receptor Mediates the Growth-Inhibitory Effect of Retinoic Acid by Promoting Apoptosis in Human Breast Cancer Cells. Mol. Cell Biol. *16*, 1138.

50. Bernadette, B., Kirton, L., Marec-Bérard, P., Gaspar, N., Laurence, V., Martín-Broto, J., Gelderblom, H., Owens, C., Anderton, J., Strauss, S.J., et al. (2026). Addition of zoledronic acid to consolidation chemotherapy in Ewing sarcoma—EURO EWING 2012 (EE2012): an international, open-label, randomised controlled phase III trial. Br. J. Cancer *134*, 1393–1402. https://doi.org/10.1038/s41416-026-03367-4.

51. Mahlendorf, D.E., and Staege, M.S. (2013). Characterization of Ewing sarcoma associated cancer/testis antigens. Cancer Biol. Ther. *14*, 254–261. https://doi.org/10.4161/cbt.23298.

52. Samson, A., Scott, K.J., Taggart, D., West, E.J., Wilson, E., Nuovo, G.J., Thomson, S., Corns, R., Mathew, R.K., Fuller, M.J., et al. (2018). Intravenous Delivery of Oncolytic Reovirus to Brain Tumor Patients Immunologically Primes for Subsequent Checkpoint Blockade. Sci. Transl. Med. *10*, eaam7577. https://doi.org/10.1126/scitranslmed.aam7577.

53. Lovatt, C., and Parker, A.L. (2023). Oncolytic Viruses and Immune Checkpoint Inhibitors: The "Hot" New Power Couple. Cancers (Basel) *15*, 4178. https://doi.org/10.3390/cancers15164178.

54. Shen, W., Patnaik, M.M., Ruiz, A., Russell, S.J., and Peng, K.-W. (2016). Immunovirotherapy with vesicular stomatitis virus and PD-L1 blockade enhances therapeutic outcome in murine acute myeloid leukemia Key Points. https://doi.org/10.1182/blood-2015.

55. Annels, N.E., Mansfield, D., Arif, M., Ballesteros-Merino, C., Simpson, G.R., Denyer, M., Sandhu, S.S., Melcher, A.A., Harrington, K.J., Davies, B., et al. (2019). Phase I trial of an ICAM-1-targeted immunotherapeutic-coxsackievirus A21 (CVA21) as an oncolytic agent against non muscle-invasive bladder cancer. Clin. Cancer Res. *25*, 5818–5831. https://doi.org/10.1158/1078-0432.CCR-18-4022.

56. Balasubramanian, A., Marceaux, C., Kanwal, S., Tarasova, I., Batey, D., Senko, C., Asadi, K., Yokote, K., Palmer, J., Christie, M., et al. (2025). Effects of CVA21, an Oncolytic Virus, in Combination with Pembrolizumab on Immunogenicity and the Tumor Microenvironment in Advanced NSCLC: A Phase I/II Trial. Clin. Cancer Res. *31*, 4644–4654. https://doi.org/10.1158/1078-0432.CCR-25-1449.

57. Rübe, C.E., Van Valen, F., Wilfert, F., Palm, J., Schuck, A., Willich, N., Winkelmann, W., Jürgens, H., and Rübe, C. (2003). Ewing's sarcoma and peripheral primitive neuroectodermal tumor cells produce large quantities of bioactive tumor necrosis factor-α (TNF-α) after radiation exposure. Int. J. Radiat. Oncol. Biol. Phys. *56*, 1414–1425. https://doi.org/10.1016/S0360-3016(03)00418-8.

58. van Valen Kentrup-Lardong, B.T.C.R.W., Winkelmann, F.V., Jiirgens, H., Winkelmann, W., Riibe, C., and Jtirgens, H. (1997). Regulation of the Release of Tumour Necrosis Factor (TNF) and Soluble TNF Receptor by 7 Irradiation and Interferon 7 in Ewing's Sarcoma/peripheral Primitive Neuroectodermal Tumour Cells (Springer-Verlag).

59. Pokrovska, T.D., Jacobus, E.J., Puliyadi, R., Prevo, R., Frost, S., Dyer, A., Baugh, R., Rodriguez-Berriguete, G., Fisher, K., Granata, G., et al. (2020). External beam radiation therapy and enadenotucirev: Inhibition of the DDR and mechanisms of radiation-mediated virus increase. Cancers (Basel) *12*, 798. https://doi.org/10.3390/cancers12040798.

60. O'Cathail, S.M., Pokrovska, T.D., Maughan, T.S., Fisher, K.D., Seymour, L.W., and Hawkins, M.A. (2017). Combining Oncolytic Adenovirus with Radiation—A Paradigm for the Future of Radiosensitization. Front. Oncol. *7*, 153. https://doi.org/10.3389/fonc.2017.00153.

61. Jayalie, V.F., and Sekarutami, S.M. (2022). Combining oncolytic virus and radiation therapy for cancer management. J. Cancer Metastasis Treat. *8*, 17. https://doi.org/10.20517/2394-4722.2021.212.

62. Annels, N.E., Arif, M., Simpson, G.R., Denyer, M., Moller-Levet, C., Mansfield, D., Butler, R., Shafren, D., Au, G., Knowles, M., et al. (2018). Oncolytic Immunotherapy for Bladder Cancer Using Coxsackie A21 Virus. Mol. Ther. Oncolytics *9*, 1–12. https://doi.org/10.1016/j.omto.2018.02.001.

63. Skelding, K.A., Barry, R.D., and Shafren, D.R. (2009). Systemic targeting of metastatic human breast tumor xenografts by Coxsackievirus A21. Breast Cancer Res. Treat. *113*, 21–30. https://doi.org/10.1007/s10549-008-9899-2.

64. Andtbacka, R.H., Curti, B.D., Hallmeyer, S., Feng, Z., Paustian, C., Bifulco, C., Fox, B., Grose, M., and Shafren, D. (2015). Phase II calm extension study: Coxsackievirus A21 delivered intratumorally to patients with advanced melanoma induces immune-cell infiltration in the tumor microenvironment. J. Immunother. Cancer *3*, P343. https://doi.org/10.1186/2051-1426-3-s2-p343.

65. Burnett, W.J., Burnett, D.M., Parkman, G., Ramstead, A., Contreras, N., Gravley, W., Holmen, S.L., Williams, M.A., and VanBrocklin, M.W. (2021). Prior Exposure to Coxsackievirus A21 Does Not Mitigate Oncolytic Therapeutic Efficacy. Cancers (Basel) *13*, 4462. https://doi.org/10.3390/cancers13174462.

66. Harrell, F.E., Califf, R.M., Pryor, D.B., Lee, K.L., and Rosati, R.A. (1982). Evaluating the Yield of Medical Tests. JAMA *247*, 2543.

67. Burchill, S., and Roundhill, E. (2021) Total RNA sequencing of patient derived Ewing sarcoma and Ewing sarcoma CSCs. University of Leeds. [Dataset] https://doi.org/10.5518/887.

68. Burchill, S., and Roundhill, E. (2023) Total RNA sequencing of Ewing sarcoma cell lines and 3D spheroids. University of Leeds. [Dataset] https://doi.org/10.5518/1210

OMTON, Volume *34*

Supplemental information

ICAM-1 as a biomarker of efficacy for oncolytic coxsackievirus A21 in patient-derived Ewing sarcoma models

Richard T. Baugh, Tyler Barr, Elizabeth A. Roundhill, Fay Ismail, Milene Volpato, Graham P. Cook, Susan A. Burchill, and Fiona Errington-Mais

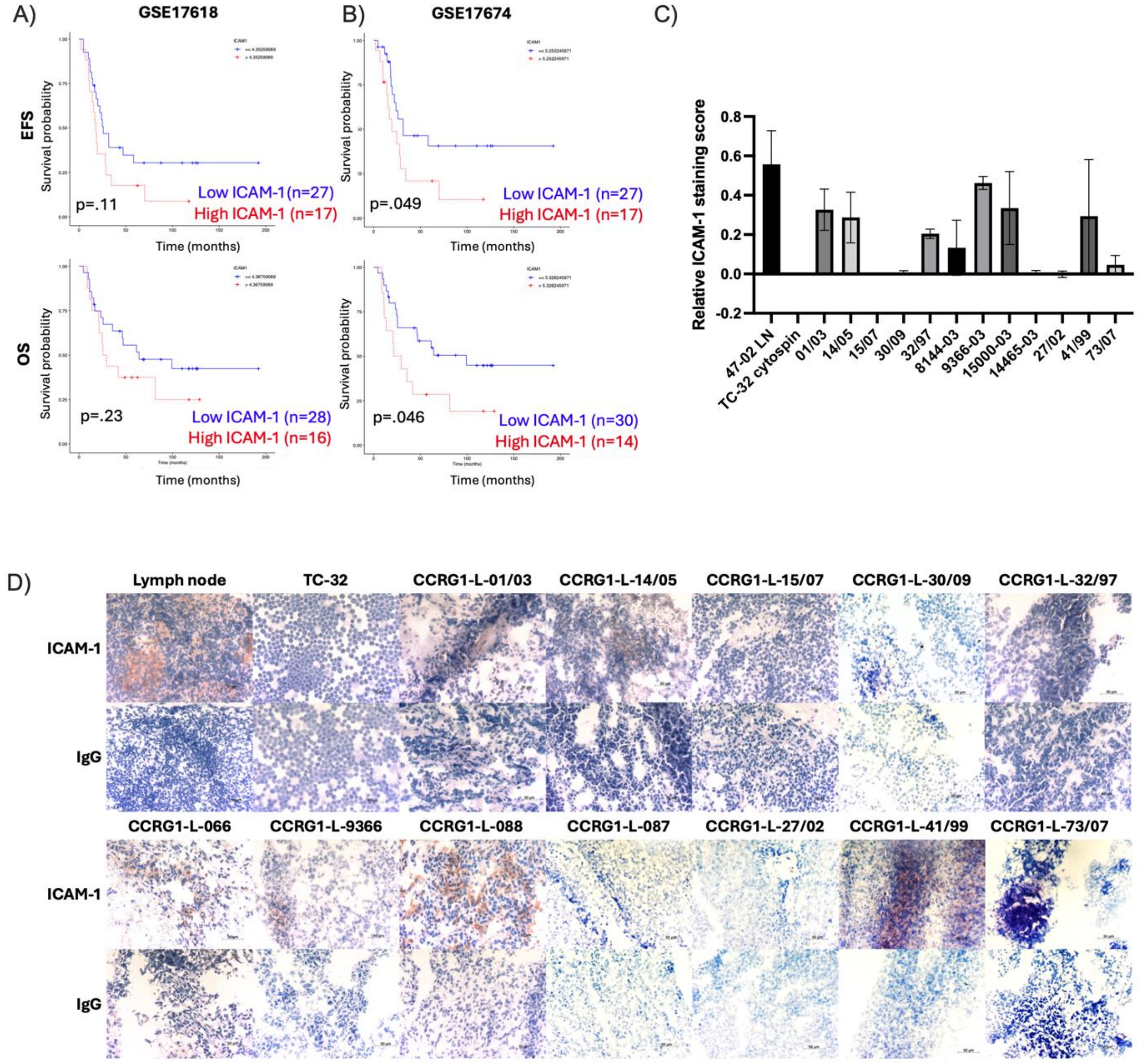

Figure S1. ICAM-1 expression in EWS. **A)** Kaplan-Meier event-free survival (EFS) and overall survival (OS) curves of high and low ICAM-1 expression from publicly available data sets GSE17618 and **B)** GSE17674. **C)** Quantification of IHC for ICAM-1. ICAM-1 staining was quantified using ImageJ analysis, with ICAM-1 score of DAB signal normalised to H&E nuclear staining for each image. N≥3 representative images taken for each section on 40x magnification. ICAM-1 scores were normalised by subtracting mean staining score from isotype-stained samples. **D)** Representative examples of ICAM-1 staining in EWS tissue by IHC. Isotype control (IgG2b) was used as a negative control for each sample. Human Lymph node tissue was used as a positive control and a cytospin of TC-32 cells used as a negative control. Representative images are shown at 40x magnification.

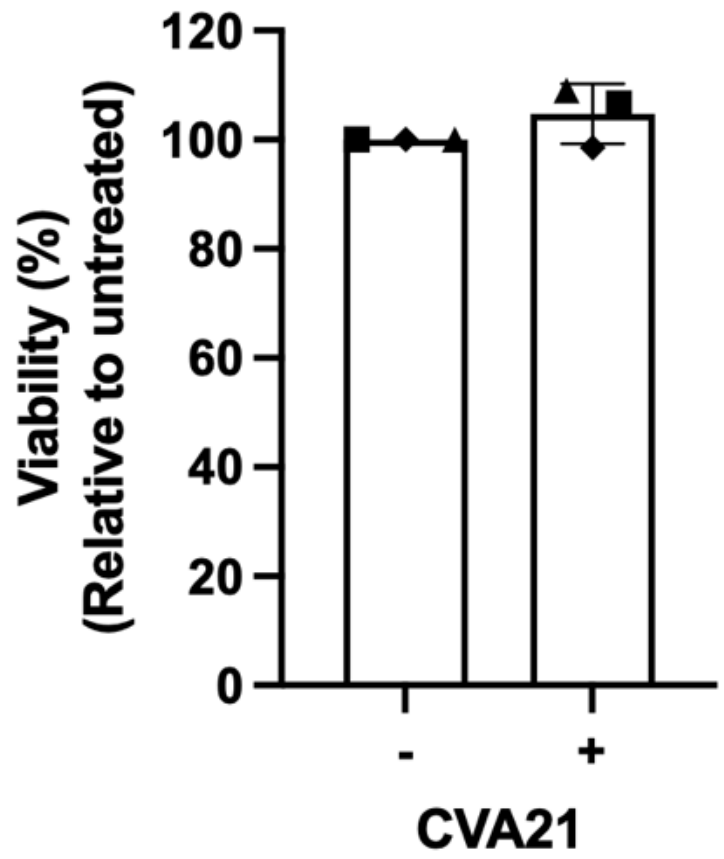

Figure S2. Short-term CVA21 infection of PDES has no oncolytic effect. A) PDES (n=3) were infected with CVA21 (MOI 2.5 – equivalent dose used in CVA21-stimulated PBMC assays; 5 hours). Cell viability was measured by CellTitre Glo and data shows the mean percentage viability of cells, relative to the untreated control (± SD). A paired t-test was performed.

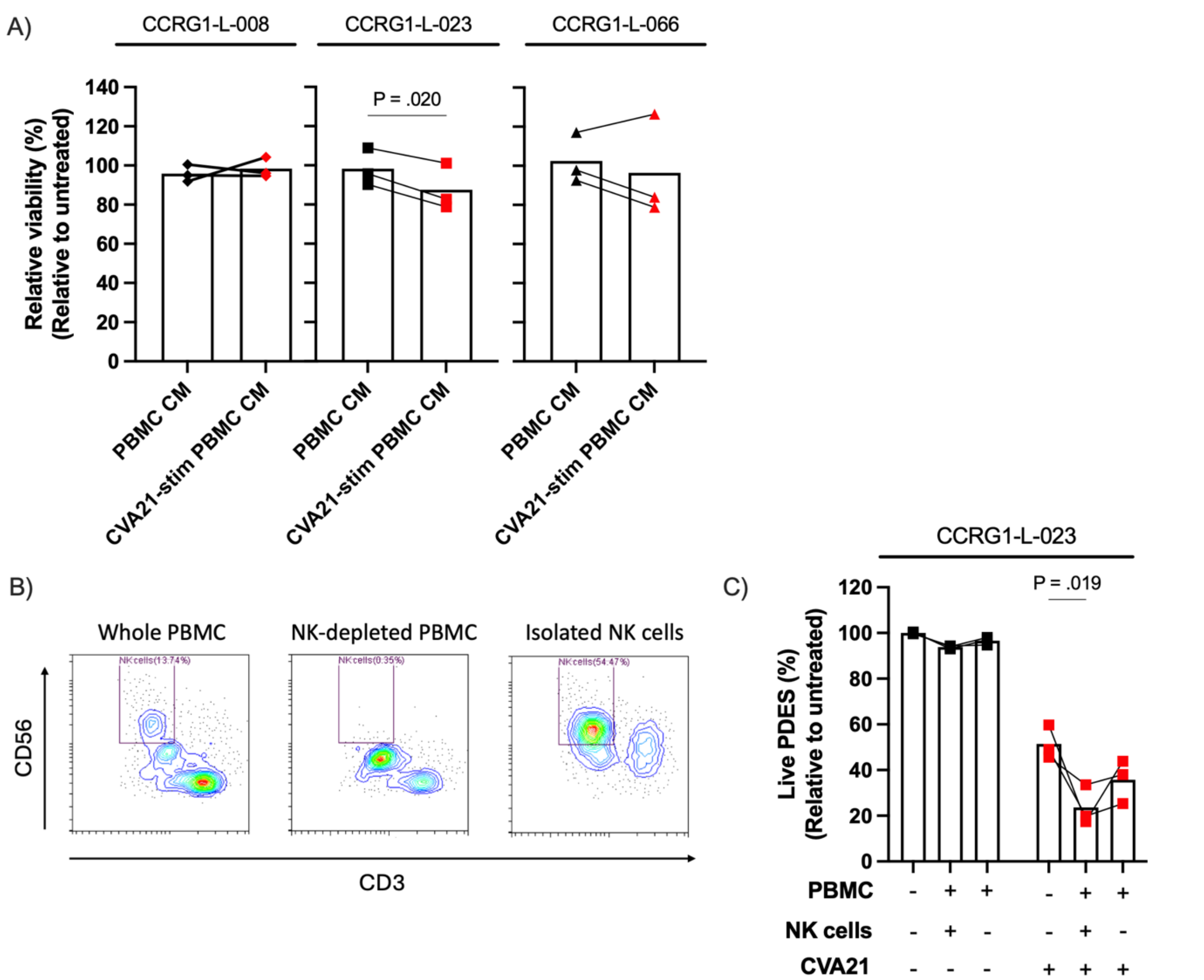

Figure S3. Mechanisms of immune-mediated killing of PDES. A) Percentage cell viability of PDES (n=3) treated with PBMC CM or CVA21-stimulated PBMC conditioned media (1:1 RPMI:CM). For PBMC CM, n=3 donors; CVA21 stimulation with MOI 0.1 for 48 hours. Cell viability was measured by CellTitre Glo 48 hours post treatment. Data is presented as mean percentage viability of cells relative to the untreated control (± SD). Paired t-tests were performed. **B)** Representative flow cytometry plots following NK cell depletion from whole PBMC. PBMC were stained with anti-CD45, anti-CD56 and anti-CD3. Plots represent live $CD45^+$ population. **C)** Percentage live PDES cells infected with CVA21 alone, or in co-culture with whole PBMC or NK-depleted PBMC. Adherent PDES were infected (or left untreated) with CVA21 (MOI 0.5) for 2 hours before co-culture with unstimulated PBMC ± NK cells for 48 hours (n=1; n=3 PBMC donors) at a 10:1 PBMC: PDES ratio. Cell viability was measured using LIVE/DEAD flow cytometry and data is presented as mean percentage live PDES ($CD45^-$) relative to

PDES in the absence of PBMC; each line represents a different PBMC donor. A two-way ANOVA with Šídák's post-hoc test was performed.

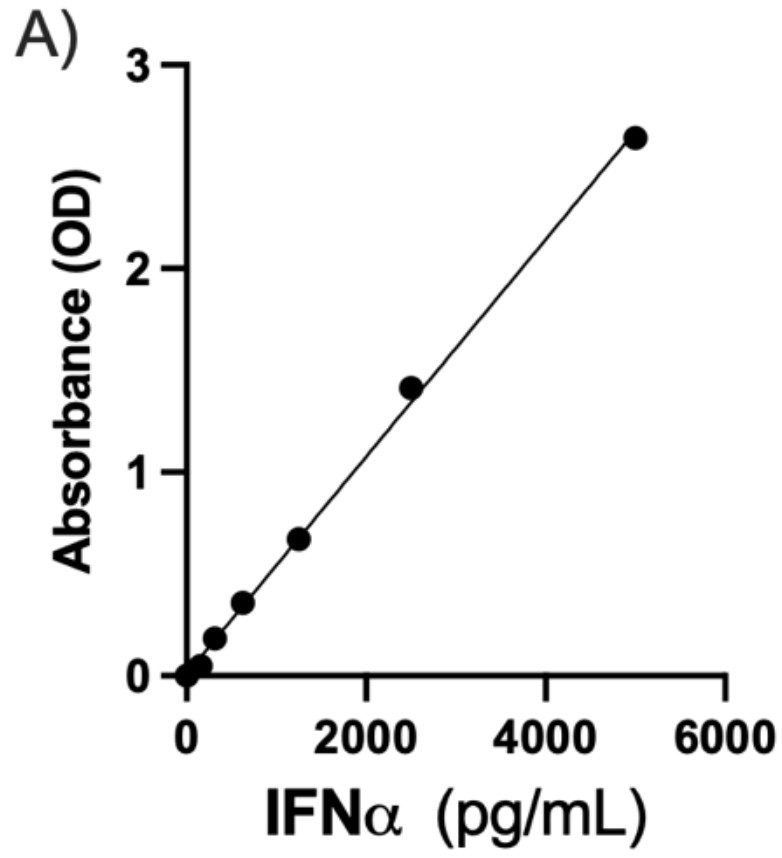

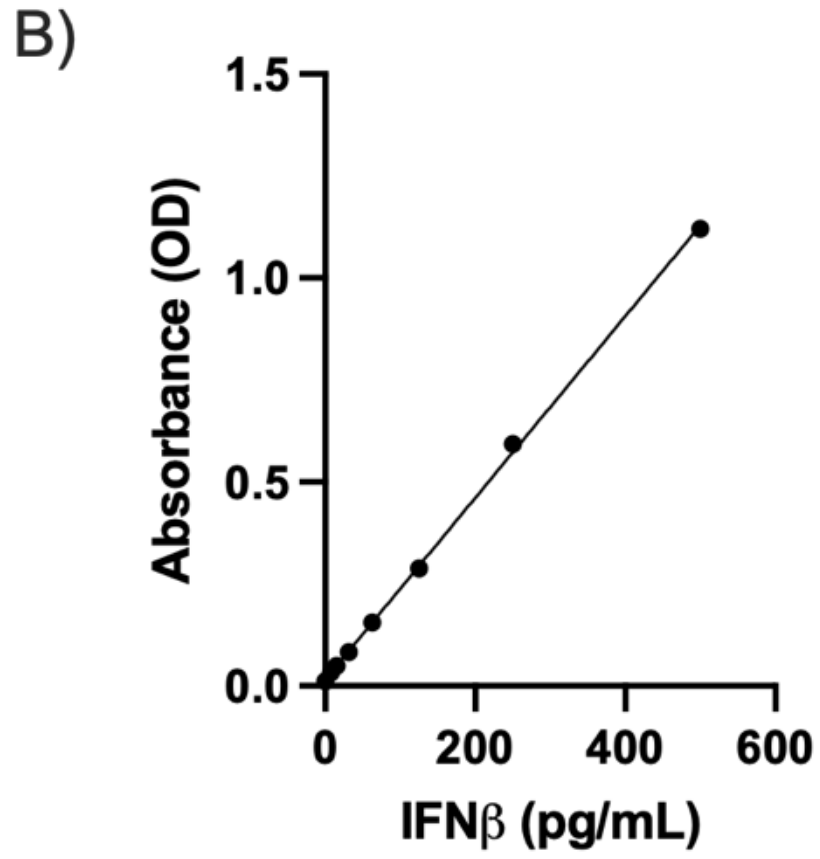

IFNα mean normalised OD values

	PDES		
**MOI**	**CCRG-L-017**	**CCRG-L-023**	**CCRG-L-066**
**0**	-98.67	-58.00	-111.33
**0.1**	-83.33	-98.00	-106.00
**1**	-107.33	-78.00	-122.67

IFNβ mean normalised OD values

	PDES		
**MOI**	**CCRG-L-017**	**CCRG-L-023**	**CCRG-L-066**
**0**	0.07	0.03	0.03
**0.1**	0.07	0.06	0.03
**1**	0.07	0.06	0.04

Figure S4. PDES do not produce type I IFN in response to CVA21. **A)** Standard curves and normalised optical density (OD) values from IFNα and **B)** IFNβ ELISAs. PDES (n=3) were infected with CVA21 at MOI 0, 0.1 and 1 for 48h before supernatant was collected for ELISA. OD readings normalised by subtracting blank well OD values. Data represents mean normalised OD from n=2 technical repeats.

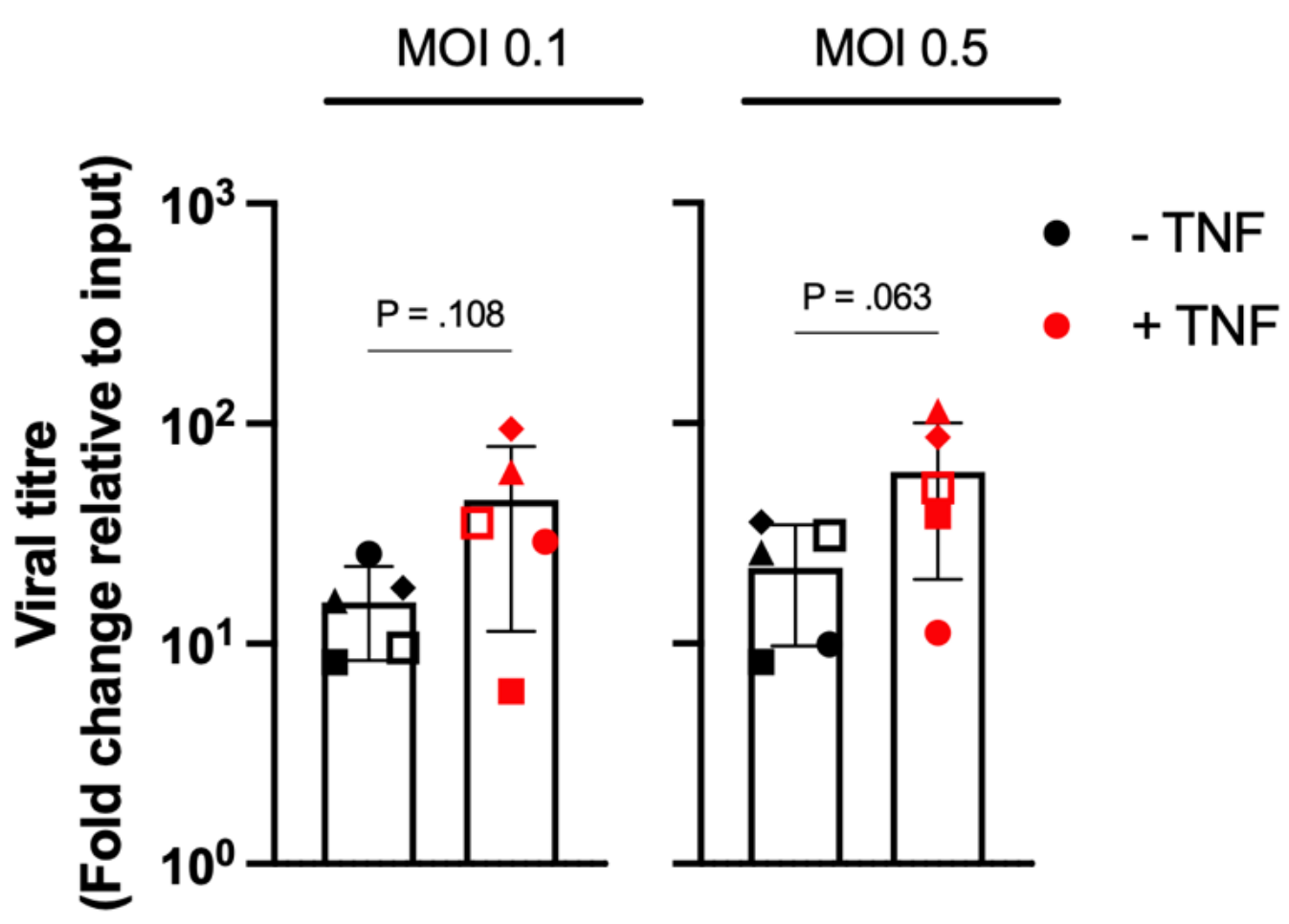

Figure S5. Fold change in CVA21 viral titre after PDES (n=5) were pre-treated with ± TNFα (5 ng/mL; 24 hours) before infection with CVA21 (MOI 0.1 and 0.5). Total cell supernatant and lysates were collected 16h post infection and CVA21 titres were measured by plaque assay. Mean fold change in viral titre compared to input virus titre for each PDES is shown. A paired t-test was performed.

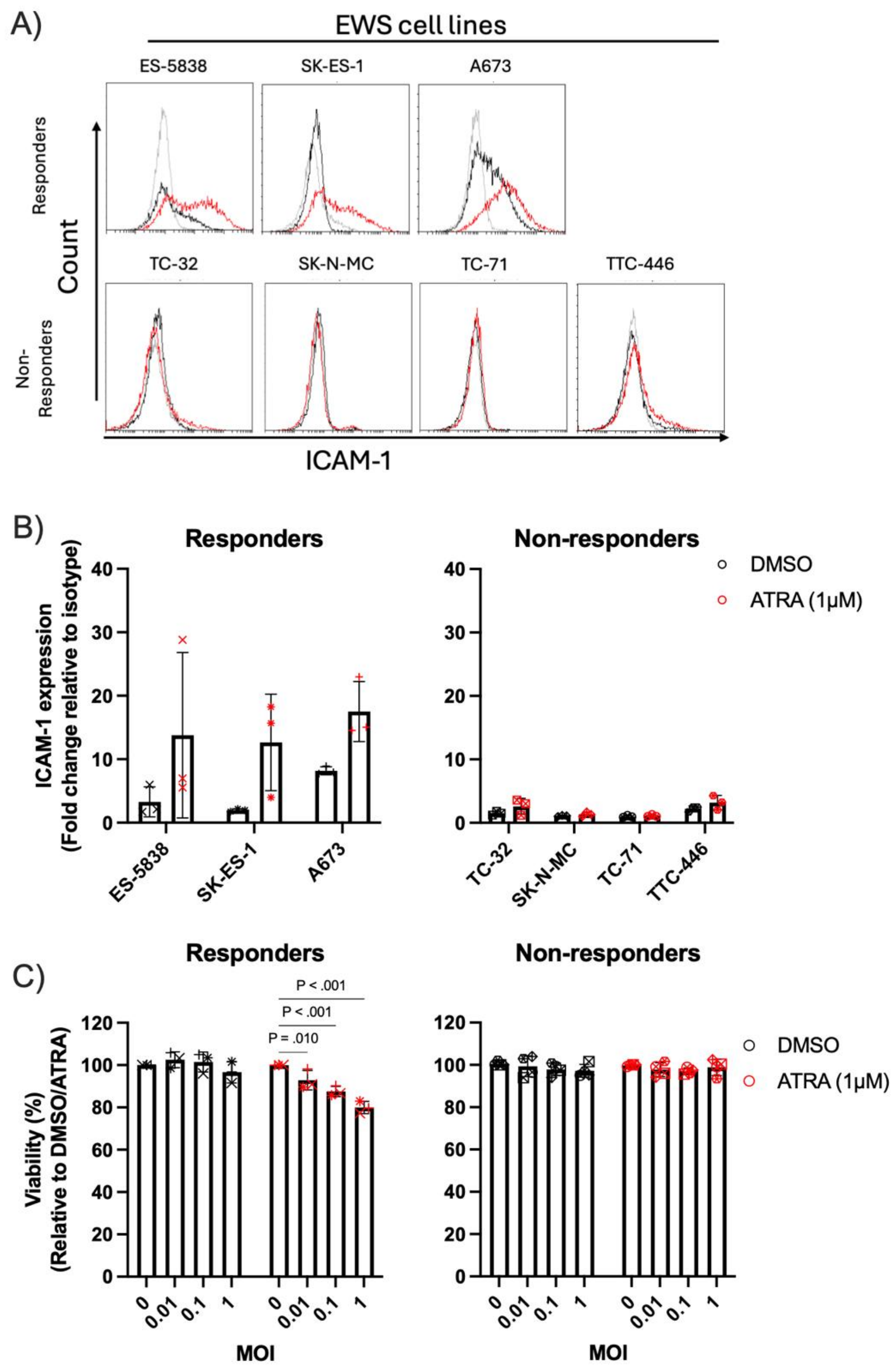

Figure S6. EWS cell lines display heterogeneous responses to ATRA-induced ICAM-1 expression and CVA21 susceptibility A) Representative flow cytometry histograms of EWS cell line surface ICAM-1 expression on control (DMSO; 72 hours; black line) and ATRA treated (1 μM; 72 hours; red line) cells compared to isotype control (grey line). **B)** Relative surface ICAM-1 expression on EWS cell lines (n=7) after treatment described in A). EWS cell lines were grouped as responders (ES-5838, SK-ES-1 and A673) and non-responders (TC-32, SK-N-MC, TC-71 and TTC-446). A two-way ANOVA with

Šídák's multiple comparisons test was performed. **C)** Percentage cell viability of EWS cell lines (n=7) following pre-treatment described in A) before infection with increasing doses of CVA21. Cell viability was measured by CellTitre Glo 48h post-infection. Data shows the mean percentage viability of cells relative to the DMSO or ATRA alone control (± SD). EWS cell lines were grouped as responders and non-responders in terms of ICAM-1 upregulation in A). A Two-way ANOVA with Dunnett's multiple comparisons test was performed.

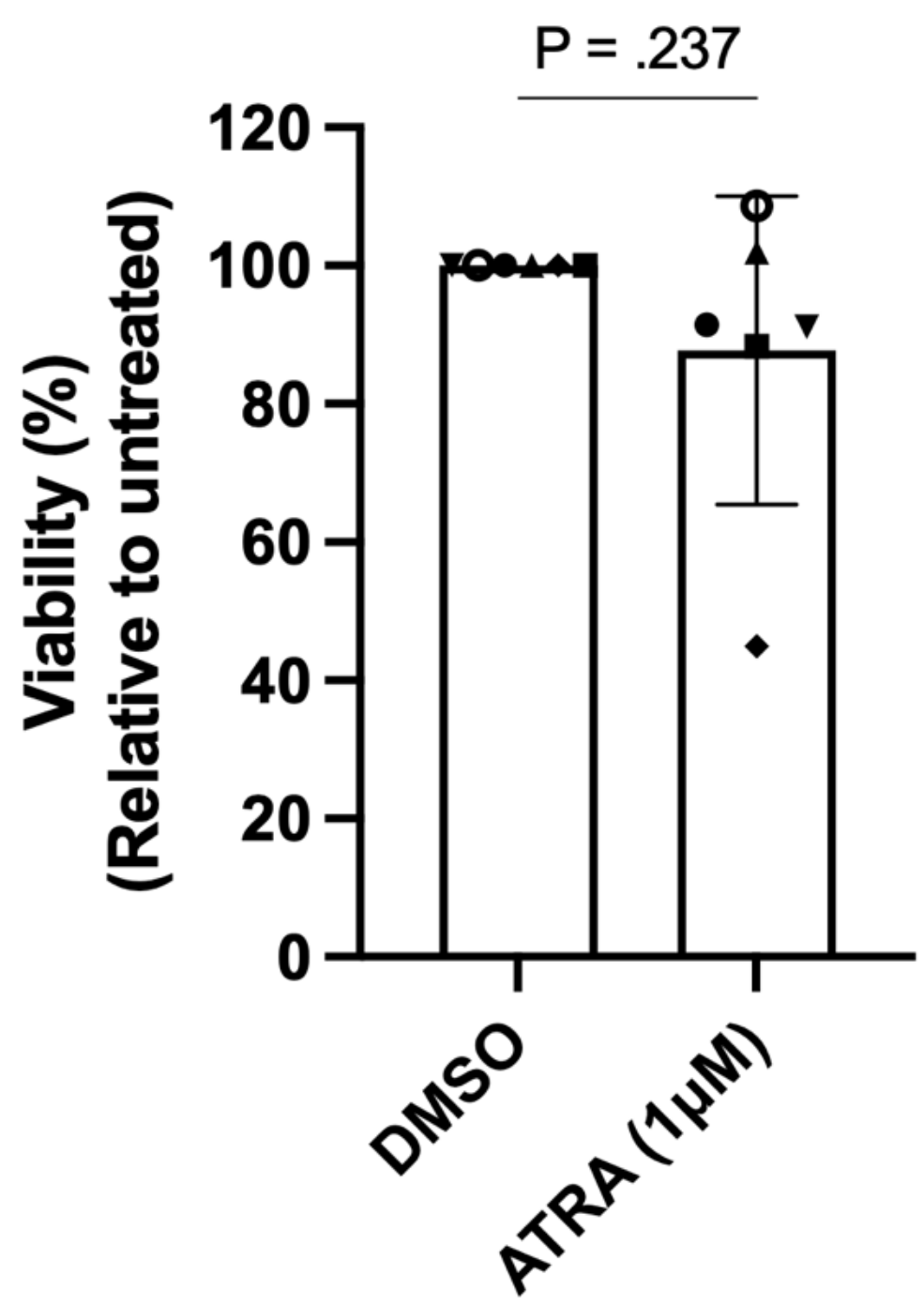

Figure S7. Cytostatic effects of ATRA on PDES. Relative viability of PDES (n=6) following treatment with 1 µM ATRA or DMSO for 72h. Cell viability was measured by CellTitre Glo 72 hours post treatment. Data is presented as mean percentage viability of PDES relative to the DMSO control. A paired t-test was performed.

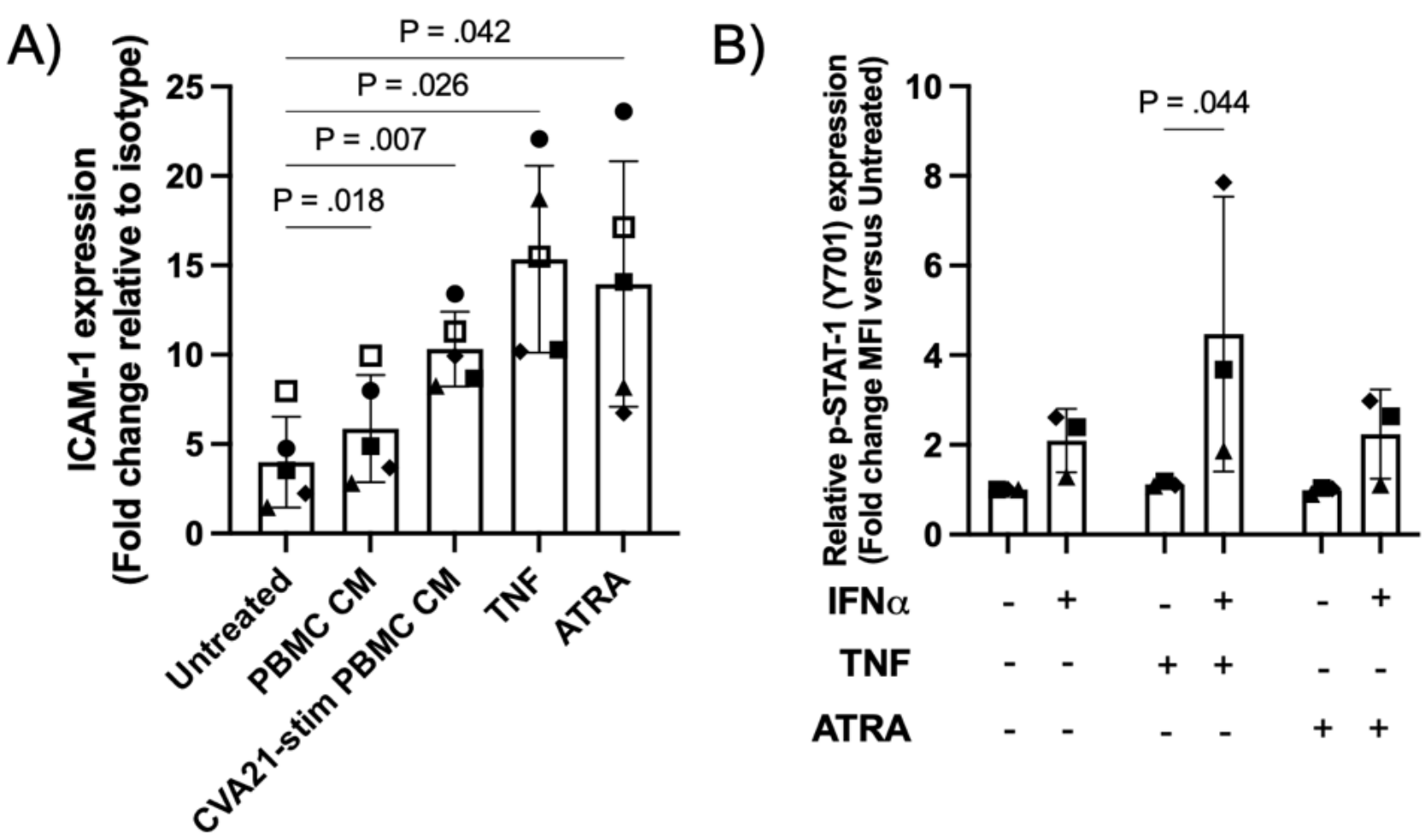

Figure S8. **Relative PDES surface ICAM-1 and intracellular phospho-STAT1 expression with different pre-treatment strategies**. **A)** Fold change in PDES (n=5) surface ICAM-1 expression following treatment with PBMC conditioned media (PBMC CM; 1:1 RPMI:CM; 24h), CVA21-stimulated PBMC conditioned media (CVA21-stim PBMC CM; 1:1 RPMI:CM; 24h), TNF (5 ng/mL; 24h) or ATRA (1μM; 72h). Data represents mean fold change in ICAM-1 expression relative to untreated control cells. PBMC CM was collected from n=3 individual donors. A one-way ANOVA with Dunnett's multiple comparisons test was performed. **B)** Fold change intracellular pSTAT-1 expression following pre-treatment with TNF (5 ng/mL; 24h) or ATRA (1μM; 72h) before treatment with IFNα (5 ng/mL; 30 min). Data represents mean fold change in pSTAT-1 expression relative to isotype control from n=3 PDES. A two-way ANOVA with Šídák's multiple comparisons test was performed.

Table S1. Summary of samples stained for ICAM-1 immunohistochemistry

**Sample**	**Quality**	**ICAM-1 detected**	**Relative ICAM-1 score (±S.D)**	**Notes**
Lymph node	+	+	0.56±0.17	Positive control
TC-32	+	-	0.00±0.00	Negative control
CCRG1-L-01/03	+	+	0.33±0.11	
CCRG1-L-14/05	+	+	0.29±0.13	
CCRG1-L-30/09	+	-		
CCRG1-L-32/97	+	-	0.20±0.02	
CCRG1-L-066	+	+	0.13±0.14	Matched PDES
CCRG1-L-9366	+	+	0.46±0.03	
CCRG1-L-088	+	+	0.33±0.19	Relapsed tumour<br>Matched PDES
CCRG1-L-087	+	-	0.01±0.01	Relapsed tumour<br>Matched PDES
CCRG1-L-27/02	+	-	0.00±0.02	
CCRG1-L-41/99	+	+	0.29±0.29	
CCRG1-L-73/07	+	-	0.05±0.05	
CCRG1-L-023	-	N/A		Poor tissue quality
CCRG1-L-075	-	N/A		Poor tissue quality
CCRG1-L-35/04	-	N/A		High non-specific staining
CCRG1-L-72/03	-	N/A		High non-specific staining
**Total**	**11/15**	**6/11**		
